# A novel statistical feature selection framework for biomarker discovery and cancer classification via multiomics integration

**DOI:** 10.1186/s12874-025-02713-z

**Published:** 2025-12-17

**Authors:** Moshira S. Ghaleb, Maryam N. Al-Berry, Hala M. Ebied, Mohamed F. Tolba

**Affiliations:** Scientific Computing Department Faculty of Computer and Information Sciences, Ain Shams University, Cairo, Egypt

**Keywords:** TCGA, SDCFE, Machine learning, Multiomics, RNA-seq, Methylation, LUSC, Statistical, Pancancer, Cancer stage

## Abstract

**Background:**

Early cancer diagnosis is essential for improving prognosis and guiding treatment. However, the high dimensionality and complexity of omics data present major challenges. Computational approaches that extract stable biomarkers and enable reliable classification across cancer types and stages are needed.

**Methods:**

A novel feature selection method, sDCFE (synergistic Discriminative Cluster-based Feature Extraction), was developed by extending Fisher-like variance analysis with a median absolute deviation (MAD) regularization term and a cluster separation component to enhance robustness and interpretability. Features selected by sDCFE were compared with those obtained from XGBoost, and the intersected set of 82 genes was evaluated through functional enrichment (KEGG, Reactome, GO BP), survival analysis (Kaplan–Meier, Cox regression), and biomarker novelty assessment against six external resources. Hybrid classification models integrating XGBoost, sDCFE, and deep learning were applied to pancancer classification, and the framework was further extended to lung squamous cell carcinoma (LUSC) staging using RNA-seq and methylation data.

**Results:**

The overlap between sDCFE and XGBoost yielded 82 candidate biomarkers enriched in cancer-related pathways, including cell cycle regulation, immune signalling, and DNA repair. Novelty assessment stratified these genes into established, emerging, and novel categories. Six genes—HFE2, LOC339674, SERINC2, SFTA3, SOX2OT, and ACPP—emerged as the most promising candidates, supported by enrichment and survival associations across multiple cancers. The hybrid model achieved near-perfect pancancer classification on TCGA (accuracy = 99.3%, MCC = 0.992, AUC = 1.0) and demonstrated strong generalizability on PCAWG (accuracy = 94%, MCC = 0.929, AUC = 0.997). In the LUSC staging task, multiomics integration improved classification performance: the CNN-based model reached 84% accuracy, while logistic regression applied to sDCFE-ranked features achieved 88.5% accuracy with superior calibration, highlighting the robustness of the selected features.

**Conclusion:**

sDCFE provides a principled extension of Fisher-like methods, enabling stable and interpretable biomarker selection. When combined with XGBoost and deep learning, the framework achieves highly accurate and biologically grounded cancer classification across both cancer types and stages. The identification of novel and prognostic biomarkers, including HFE2, LOC339674, SERINC2, SFTA3, SOX2OT, and ACPP, underscores its translational potential. These results position the framework as a promising precision oncology tool to support early diagnosis, risk stratification, and treatment decision-making.

**Supplementary Information:**

The online version contains supplementary material available at 10.1186/s12874-025-02713-z.

## Background

 Cancer is regarded as one of the leading causes of mortality worldwide, with millions of new cases being diagnosed each year. The early and accurate detection of cancer types and stages is considered essential for the improvement of survival outcomes and the tailoring of personalized treatment strategies. Traditional diagnostic procedures—such as histopathology and imaging—although effective, are often regarded as invasive and insufficient for capturing the molecular heterogeneity underlying cancer progression [1–3]. With the advent of high-throughput sequencing technologies such as RNA-seq, large-scale molecular profiling has been enabled, allowing the discovery of biomarkers [4]and the development of computational models for classification and staging [5].

Classical statistical methods (e.g., variance filtering, limma, t-tests) continue to be applied for the analysis of high-dimensional omics data, but their usefulness is limited by assumptions of linearity, sensitivity to noise, and redundancy across correlated features [6]. These constraints are often associated with unstable results across datasets, which complicates biological interpretation. By contrast, machine learning (ML)–based approaches such as XGBoost, random forests, and Lasso have been widely adopted for feature selection in pancancer studies [7]. XGBoost, in particular, has been frequently applied for ranking features by importance, but its reliance on tree splits has been shown to produce unstable rankings, overfitting, and limited interpretability in high-dimensional omics. For example, strong accuracy was reported by Almuayqil et al. [8] when CNN models were trained on XGBoost-derived features, yet the reproducibility of gene rankings was shown to be uncertain. Similarly, intersecting biomarkers identified through statistical and ML pipelines were observed by Ghaleb et al. [9] to have low overlap, raising concerns regarding consistency. Comparable concerns about instability in tree-based importance measures have also been reported in other studies [10, 11]. Although XGBoost is regarded as one of the most widely used and effective feature selection methods in bioinformatics, its limitations in stability and interpretability are considered to necessitate complementary approaches [12]. These challenges indicate that a methodological gap is present: the balance between statistical rigor and discriminative power required for reproducible biomarker discovery in noisy, heterogeneous multiomics datasets has yet to be achieved.

Recent developments have been characterized by the extension of ML with deep learning (DL) models for the improvement of cancer type classification and staging. DL has been increasingly utilized in medical research, and meaningful improvements in classification and decision-support applications have been demonstrated [13–16]. Pretrained CNN architectures (InceptionV3, Xception, EfficientNetB7) were employed by Hanan et al. [17] to predict drug response from omics data converted into image representations, while comparative performance of SVM, KNN, Naïve Bayes, LR, RF, DT, XGBoost, and ANN for breast cancer detection was demonstrated by Patil et al. [18], with the SVM achieving the highest accuracy. An accuracy of 99% in stage classification with deep learning was achieved by Ghaleb et al. [19] using TCGA-LUAD mRNA data, and enrichment analysis of differentially expressed genes was additionally performed. The integration of multiomics data for survival prediction in liver cancer was reported by Chaudhary et al. [20], while a DL framework for cancer classification and driver gene identification was developed by Zeng et al. [21]. Collectively, the predictive power of ML/DL has been demonstrated, yet a critical limitation remains: most pipelines are dependent on either black-box feature extraction or unstable statistical/ML pre-filters, which leaves interpretability unresolved.

Multiomics integration has been recognized as a powerful approach to address cancer complexity, as genomics, transcriptomics, proteomics, methylation, and metabolomics can be combined within a unified analytical framework [22]. A more comprehensive view of cancer progression can thus be provided, with cross-layer interactions being revealed that are overlooked by single-omics methods. Its promise has been demonstrated in numerous studies: Zhao et al. [23] integrated ncRNAs, miRNAs, and mRNAs with SVMs, achieving 95.3% accuracy; Fan et al. [24]

applied an SVM-based 12-gene signature with 91% accuracy; and Alina et al. [25] developed hybrid DL classifiers integrating expression, methylation, and clinical data, achieving high accuracy in stage definition. Building on this direction, multiomics-based classification has been extended in this study to lung squamous cell carcinoma (LUSC), in which early-stage detection is regarded as clinically critical yet highly challenging.

To address the gap observed between statistical and ML-based feature selection, sDCFE (synergistic Discriminative Cluster-based Feature Extraction) was proposed. In sDCFE, statistical variance analysis is combined with cluster separation so that the selected genes are ensured to be predictive, interpretable, and reproducible. Unlike purely statistical methods, sDCFE has been shown to be more robust to noise and redundancy, and unlike ML approaches such as XGBoost, greater emphasis is placed on the stability and interpretability of the selected features. In this study, sDCFE was integrated with XGBoost, allowing the strong predictive capacity of XGBoost to be leveraged while its limitations in stability and biological interpretability were addressed. When applied across ten pancancer datasets and validated in LUSC stage classification using RNA-seq [26]and methylation data, this integrated framework was demonstrated to hold strong potential for reproducible biomarker discovery, early cancer detection, and improved patient stratification.

This study is expected to contribute to ongoing efforts in cancer research and diagnostics by offering a potential tool for improved early detection and personalized treatment strategies. The structure of this paper is organized as follows: Section one provides background information; Section two presents the proposed models; Section three outlines the experimental results; Section four discusses the findings; and Section five concludes the work.

## Method

**Fig. 1:**
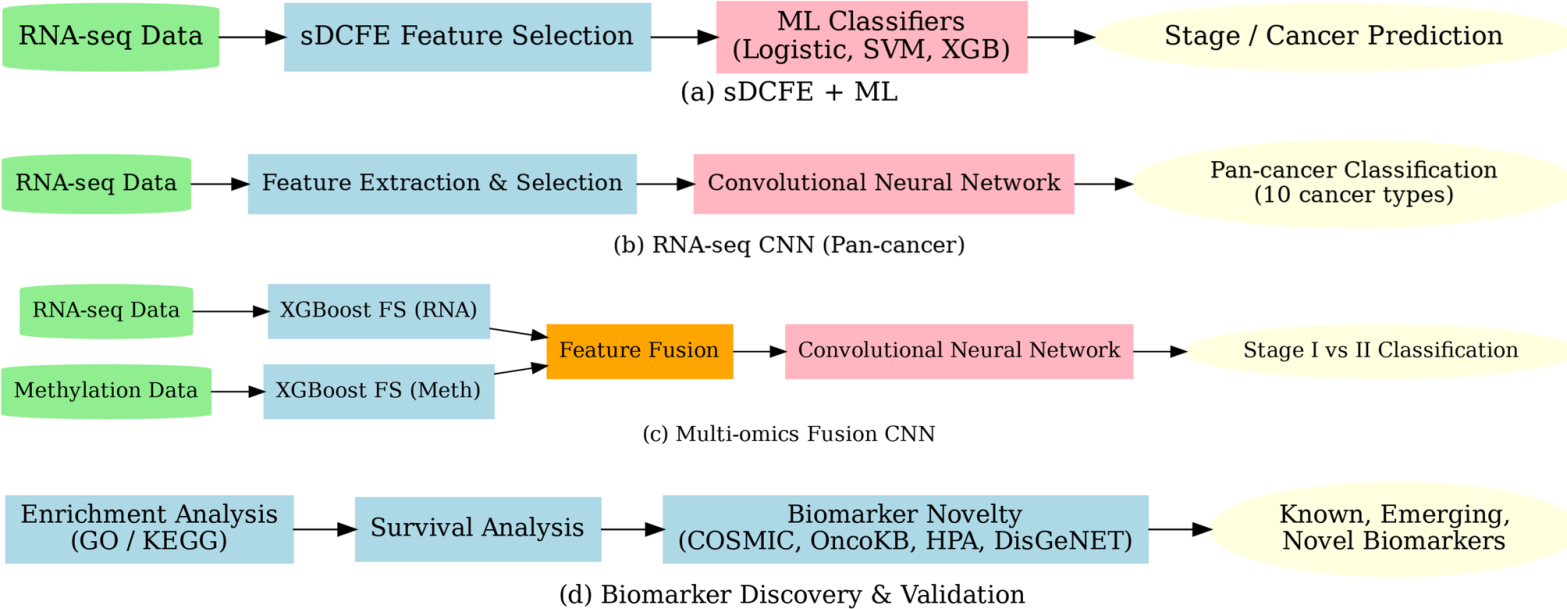
Workflow overview of the proposed study and biomarker discovery

In this work, three complementary models were developed to address the challenges of cancer classification and staging Fig. 1. All models were built on RNA-seq or multiomics data, with the aim of improving predictive performance and supporting biomarker discovery. The workflow begins with a preprocessing phase in which RNA-seq and methylation data were cleaned to remove inconsistencies, filtered to retain highly variable genes, normalized to correct technical biases, and imputed to resolve missing values. These steps ensured consistent accuracy across samples and provided reliable input features for downstream analysis.

**Fig. 2:**
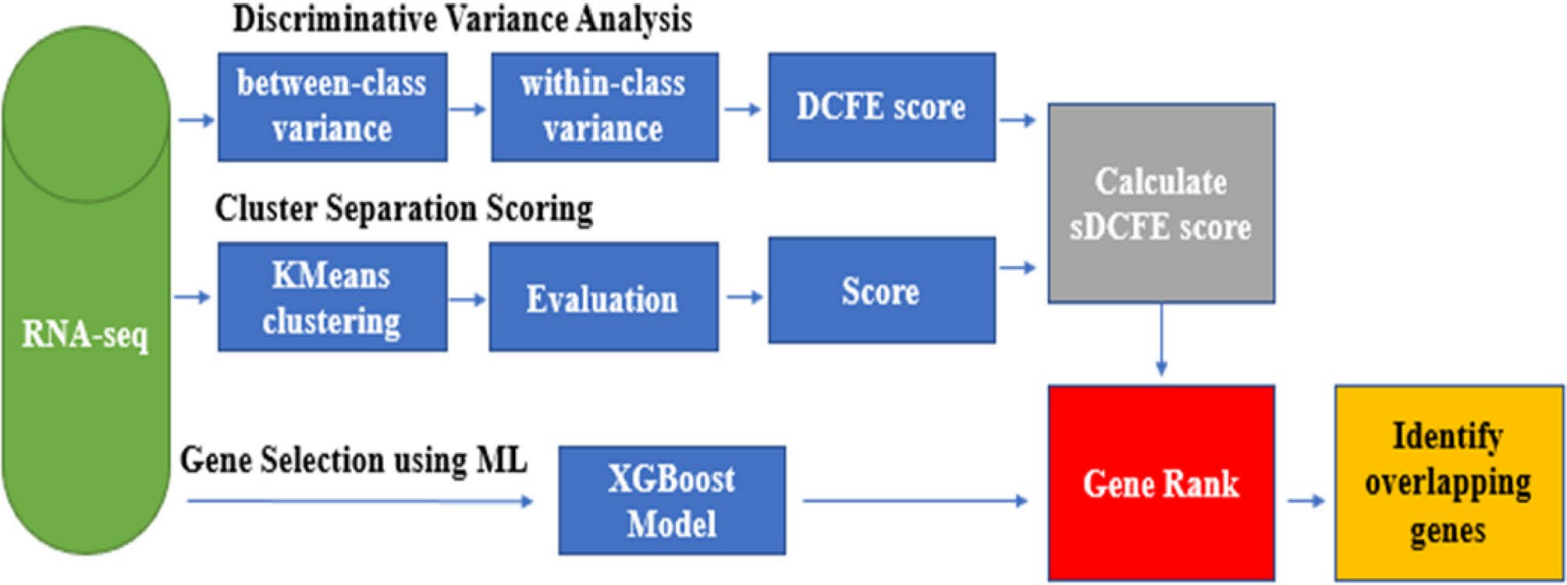
Workflow of feature selection and intersection analysis via sDCFE and XGBoost

For the first workflow Fig. 1.a, synergistic Differential Cluster Feature Extraction (sDCFE) was applied to RNA-seq data to rank genes by discriminative power. The top-ranked features were then used with conventional machine learning classifiers, including Logistic Regression, SVM, and XGBoost, for stage and cancer-type prediction. The second workflow Fig. 2.b focused on pancancer classification across ten tumors types. After preprocessing, RNA-seq data underwent feature extraction and gene selection to reduce dimensionality and highlight discriminative signals. These selected features were then used to train a Convolutional Neural Network (CNN), which captured complex nonlinear dependencies and improved multi-class classification performance. The third workflow Fig. 1.c employed a hybrid multiomics fusion approach, where RNA-seq and methylation data were separately processed with XGBoost feature selection, and the resulting reduced feature sets were merged at the feature level. A CNN was then trained on the fused features to perform binary classification of Stage I versus Stage II lung squamous cell carcinoma (LUSC). Beyond classification, the study emphasized biomarker discovery; the fourth workflow Fig. 1.d. The features selected by sDCFE, and XGBoost were systematically evaluated for biological relevance. Functional enrichment analysis (KEGG, Reactome, and GO) was conducted to uncover pathways associated with the identified genes, while survival analysis validated their prognostic significance. Biomarker novelty was assessed by comparing against established cancer-related databases, distinguishing between previously known, emerging, and novel candidates.

Together, these workflows demonstrate the effectiveness of combining statistical feature selection, machine learning, and deep learning to achieve high accuracy in cancer classification and staging, while simultaneously advancing the discovery of robust and potentially novel biomarkers with strong biological and clinical significance.

### Preprocessing phase

The preprocessing phase was applied to both pancancer classification and Lung cancer stage classification, ensuring that the data were prepared consistently and effectively for each model’s unique architecture. Several preprocessing steps were used to prepare the data for classification:

1. *Data cleaning and filtration*: Initially, data cleaning was conducted to ensure quality. Features with zero variance across all samples were removed to eliminate redundant or irrelevant information. This filtration process allowed a focus on features exhibiting variability, which were likely to be more informative for classification.
2. *Handling missing data*: The missingness rate was calculated to understand the extent of missing values within the dataset. To address this, missing values were imputed via the k-nearest neighbours (KNN) algorithm [27], which estimates missing values on the basis of sample similarity, thus maintaining dataset integrity.
3. *Normalization*: Following imputation, normalization was performed to standardize the data. A logarithmic transformation [28] was applied to reduce skewness and improve the data distribution. The data were then scaled [29] to ensure that all the features contributed equally to the model training process.
4. *Matching samples with clinical data*: The processed samples were matched with corresponding clinical data, with a specific focus on stage information. This step targeted Stage One and Stage Two data, aligning each sample with its relevant clinical stage to facilitate accurate classification.

### Feature selection phase

Feature selection is considered a critical step in pancancer and cancer stage classification, particularly when high-dimensional RNA-seq and methylation datasets are involved. Model-based approaches, such as XGBoost, have been widely adopted for feature selection because of their ability to capture complex, nonlinear interactions between features during classifier training. In this study, XGBoost was initially utilized to classify 10 different cancer types on the basis of pancancer RNA-seq data, as well as to classify lung cancer stages on the basis of multiomics integration, RNA-seq and methylation data, where high predictive accuracy was achieved.

Although XGBoost provides strong classification performance, its feature selection process is inherently model-dependent and often lacks biological interpretability. In contrast, statistical methods that prioritize genes on the basis of intrinsic class-separability properties are considered more transparent and biologically grounded alternatives. To address this limitation, a novel synergistic combination of unsupervised clustering and supervised learning signals for feature selection method, **s**DCFE, is proposed, which combines statistical variance analysis with unsupervised cluster separation scoring.

Although several traditional statistical feature selection methods have been developed, the cluster structure has rarely been integrated into the ranking process, which is crucial when analysing biological datasets containing hidden subtypes or heterogeneity.

### XGBoost - based model

Model-driven feature selection was performed using XGBoost [30], a gradient boosting framework based on decision trees. RNA-seq and methylation datasets from TCGA were provided as inputs, and classification models were trained to predict cancer types and lung cancer stages. Feature importance scores were derived on the basis of the frequency and quality of the splits in which each gene was involved across the ensemble of trees. Genes with positive importance values (importance >0) were retained as candidate features for downstream analyses.

For all experiments, the XGBoost classifier was configured with the following parameters: 200 estimators, maximum tree depth of 5, learning rate of 0.1, subsample fraction of 0.8, and column subsample by tree fraction of 0.8. A fixed random state (42) was applied to ensure reproducibility, and “logloss” was used as the evaluation metric. The multi: softprob objective was employed for multi-class pancancer classification, while the binary: logistic objective was applied for LUSC stage classification.

### Synergistic- discriminative class feature extraction (sDCFE) method

A synergistic combination of unsupervised clustering and supervised learning signals for feature selection method, termed sDCFE (Synergistic Discriminative Class Feature Extraction), was developed to perform model-independent gene ranking. The method consists of two main steps.

#### Discriminative variance analysis

Each gene’s ability to distinguish between different cancer types was quantified by calculating the ratio of between-class variance to within-class variance (similar to the Fisher score), generating a DCFE score for each gene. This formulation is related to Fisher score, which also measures between-class to within-class variance. However, sDCFE extends this principle by incorporating a MAD-based regularization term to reduce the effect of highly dispersed genes and by synergistically combining a cluster separation score to capture hidden subtypes.

For each gene , the following terms were defined:

- $\mu i$ :Mean expression of gene across all samples
- $\mu_{ic}$ :Mean expression in cancer type c
- *ac*^2^:Variance in cancer type c
- *n*^c^ :Number of samples in class c
- C: Total number of cancer types (10)
- N: Total number of samples
- $$w_c=\frac{n_c}N$$ class weight
- *MAD*_i_ median absolute deviation of gene across all samples.

The DCFE score was then computed using the following formula:

1 $$DCFE_I=\frac{\sum_{c=1}^cwc\cdot\left(\mu_{ic}-\mu_i\right)^2}{\sum_{c=1}^cwc\cdot a^2+\lambda.MAD_I}$$

λ is a regularization parameter penalizing high-dispersion genes to improve stability.

#### Cluster separation scoring

To account for label noise and hidden subtypes, an unsupervised component was incorporated using K-means clustering. For each gene, a cluster separation score was computed for each gene (F-statistic across K clusters). The final sDCFE score was defined as:

2 $$sDCFE_i=a.DCFE_I+\left(1-a\right).CS_i$$

Where αis a synergy weighting factor balancing the contributions of variance-based discrimination and cluster separation.

#### sDCFE parameterization

α = 0.7 was selected after empirical testing of values from 0.5 to 0.9; this setting provided stable rankings while emphasizing discriminative variance. λ = 0.1was used to reduce dominance by highly variable genes; this value was chosen based on subsampling stability experiments. K = 10 clusters was chosen to align with the number of cancer types, providing interpretable separation. For the LUSC staging task, where the problem is binary (Stage I vs. Stage II), the cluster separation component of sDCFE was implemented with K = 2 to reflect the two clinical classes. Genes were ranked by their sDCFE scores, and the genes select using cutoff. This cutoff was chosen because cross-validation analyses demonstrated that both feature stability and classification accuracy plateaued beyond this threshold, with minimal gains from including additional genes.

### Comparative machine learning baselines

To establish comparative benchmarks, several classical machine learning models were implemented. These baselines were trained on the gene sets obtained from sDCFE or XGBoost so that their predictive performance could be evaluated relative to the proposed deep learning frameworks.

#### sDCFE alone

To assess the discriminative capacity of sDCFE independently, a nearest-centroid classification approach was applied directly to the features selected by sDCFE. The gene subsets identified by sDCFE were extracted from the RNA-seq expression profiles, and each sample representation was standardized using z-score normalization. For classification, centroids for each cancer class were derived as the mean expression profile of the training samples within that class. Test samples were classified according to the centroid to which the Euclidean distance was minimal.

#### Support vector machine (SVM)

An SVM classifier [31] was implemented with a linear kernel and default regularization settings. The model was trained on features selected by sDCFE; using sDCFE cutoff threshold, so that its performance could be assessed in comparison with the proposed hybrid frameworks.

#### Logistic regression

Logistic Regression [32] was trained with L2 regularization on the sDCFE-selected features which selected using sDCFE cutoff threshold. This model was considered a benchmark for evaluating classification performance using a simpler, interpretable linear approach.

#### XGBoost alone

As an additional baseline, the XGBoost algorithm was used not only for feature selection but also for classification, allowing its predictive capacity to be directly evaluated.

#### sDCFE + XGBoost

To further investigate the synergy between the two feature selection approaches, features obtained from sDCFE using sDCFE cutoff threshold were used as input for XGBoost model.

All baseline models were evaluated using the same fixed TCGA training and testing splits that were applied to the deep learning experiments, and comparability across approaches was thereby ensured.

### The intersected genes between the sDCFE and XGBoost feature sets

To evaluate the consistency and differences between the model-based XGBoost and statistical-based sDCFE feature selection methods, a comparative analysis was performed.

The top-ranking genes from both methods were identified, and their intersection was computed.

A Venn diagram was generated to visualize the degree of overlap between the two gene sets.

The number of shared genes (82) was considered an indicator of agreement between the methods, providing additional confidence in the biological relevance of the consistently selected features.

Although the final classification was conducted using the XGBoost-selected genes, the intersection served as a validation step to ensure that the retained features were not solely dependent on a single selection strategy. The overall workflow of the feature selection process is illustrated in Fig. 2, in which genes are independently ranked by sDCFE and XGBoost, and an intersection analysis is subsequently performed to identify common informative genes.

### Pancancer proposed model: XGBoost + sDCFE + DL

The model was developed for cancer type classification across ten different cancer types. Preprocessing steps, as previously described, were performed, including comprehensive data filtration, cleaning, computation of missingness, and imputation of missing data. Specifically, genes with minimal variance were removed so that those with significant variability could be retained.

Feature selection was carried out using both the XGBoost algorithm and the proposed sDCFE method. XGBoost was applied to identify highly discriminative features through tree-based importance, whereas sDCFE integrated statistical variance analysis with cluster separation to improve stability and interpretability. After feature selection, the overlap between the features selected by XGBoost and sDCFE was identified, and redundant genes were removed. A final non-redundant set of 937 genes from XGBoost and 350 genes from sDCFE (totaling 1,205 features) was obtained and used for model construction. These features were integrated to form the proposed hybrid framework (sDCFE + XGB + DL).

**Fig. 3:**
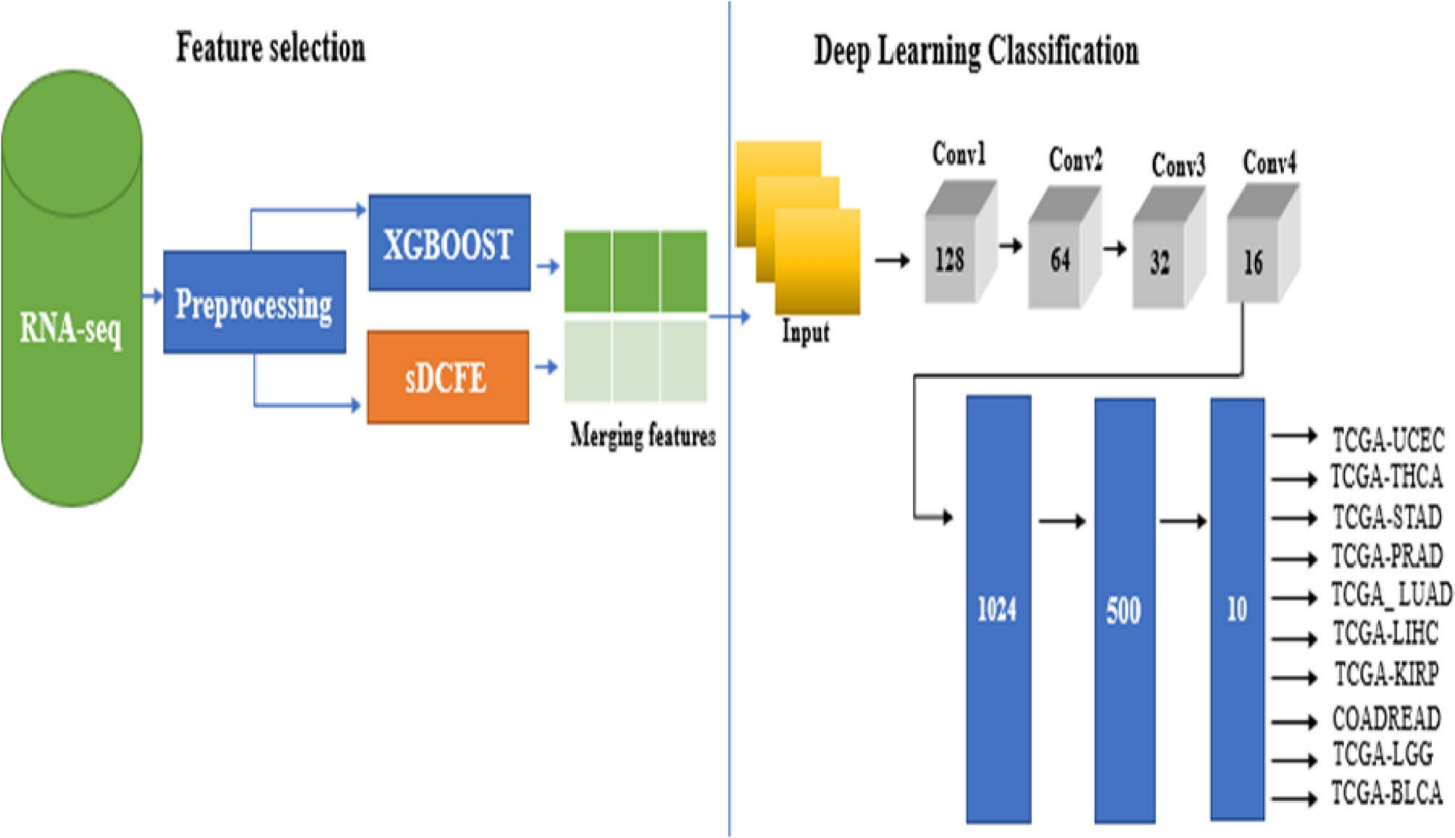
Architecture of the proposed pancancer classification model XGB + sDCFE + DL

The deep learning framework was constructed using a CNN architecture composed of four convolutional layers, a flattening layer, and three fully connected layers. The selected features were originally represented as 1D RNA-seq vectors and were subsequently reshaped into a 2D format through the NumPy *reshape* function to ensure compatibility with Conv2D layers. Each sample was therefore represented with an input dimension of (1 × 28 × 43). Convolutional kernels with sizes of 5 × 5 and 3 × 3 was empirically determined on the basis of preliminary experiments and prior studies applying CNNs to omics data, in which, despite the absence of a natural spatial structure, convolutional filters were shown to capture local feature interactions. The architecture was composed of four convolutional layers with 128, 64, 32, and 16 filters, respectively, followed by a flattening operation and three dense layers with 1024, 500, and 10 units for final classification. To reduce the risk of overfitting, dropout layers were incorporated after the dense layers and early stopping was applied during training. Through this design, XGBoost-selected features and sDCFE-selected features were jointly modelled within the CNN framework, which constituted the main proposed model for pancancer classification. Figure 3 illustrates the pancancer model 1 architecture.

For comprehensive evaluation, the proposed model was compared with several baselines: sDCFE alone, sDCFE + SVM, sDCFE + Logistic Regression, XGB alone, and sDCFE + XGB (without deep learning). This comparative design was used to assess the effectiveness of the hybrid sDCFE + XGB + DL approach against both statistical and classical machine learning pipelines. To ensure fair model assessment, the TCGA pancancer dataset was divided into fixed training and testing subsets, which were consistently used across all models. Furthermore, the PCAWG which is part of ICGC dataset was employed as an independent external validation cohort so that generalizability beyond TCGA could be evaluated.

### LUSC cancer stage proposed model

For LUSC stage classification, two complementary models were developed using the TCGA LUSC dataset. Two omics types—RNA-seq and methylation—were utilized, with preprocessing performed separately for each dataset. Feature selection was carried out on each dataset using the XGBoost algorithm, through which the most relevant features were identified (293 from RNA-seq and 628 from methylation).

#### Model 1 — late-fusion CNN model

For the LUSC stage classification task, feature extraction was first carried out separately on the RNA-seq and methylation datasets. Each dataset was processed independently to ensure high-quality inputs, after which XGBoost feature selection phase was applied to identify the most informative features from each omic type; Genes with positive importance values (importance > 0) were retained as candidate features for downstream analyses. Patients with both RNA-seq and methylation data available were then retained, and the selected features (293 RNA-seq features and 628 methylation features) were integrated through a late fusion step, yielding a combined feature set of 921 features across 307 matched samples (172 Stage I, 135 Stage II).

**Fig. 4:**
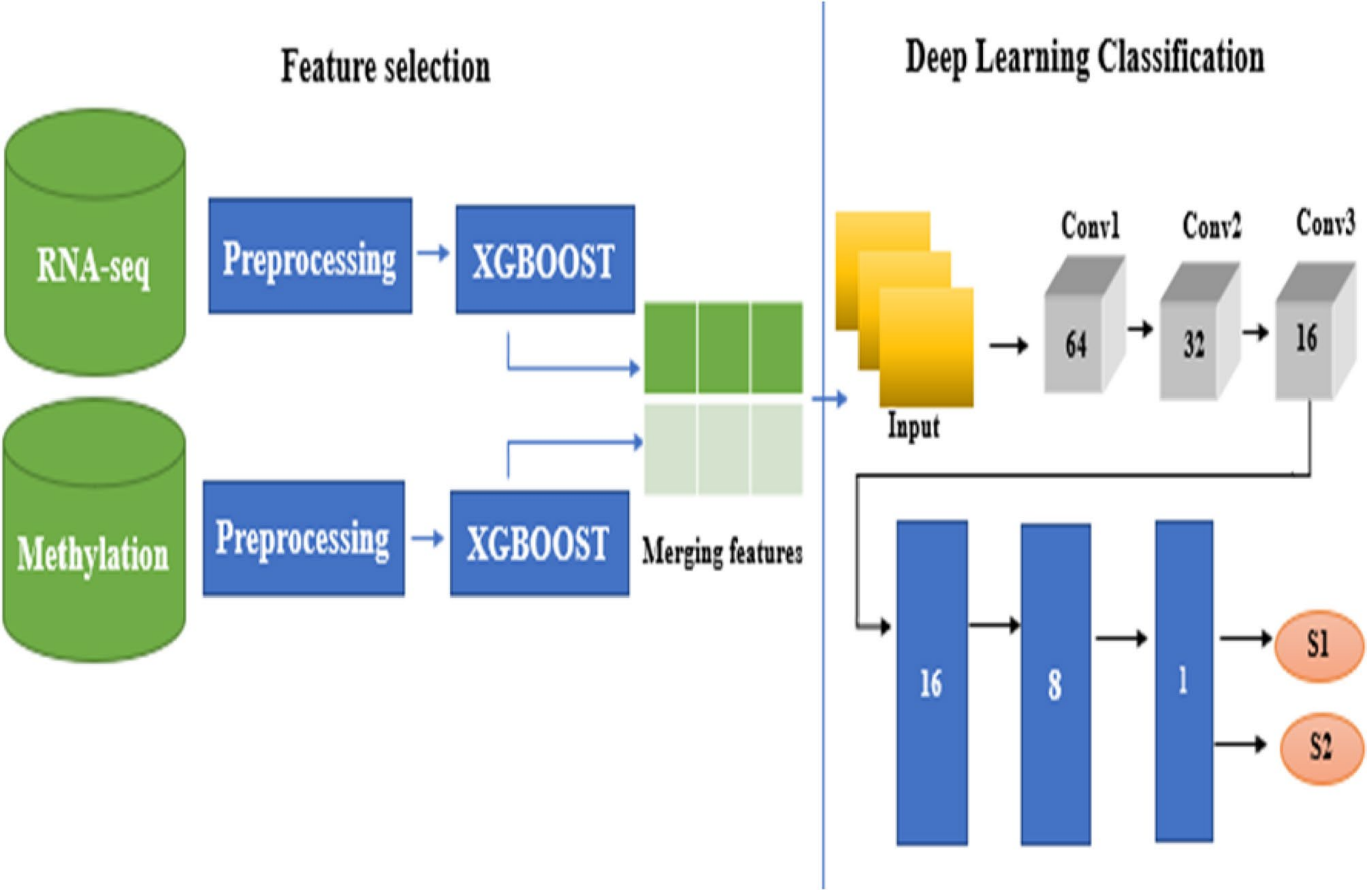
Architecture of LUSC stage classification Late fusion CNN Model

The fused feature set was subsequently reshaped into a 2D input of [1 × 23 × 40] to enable compatibility with CNN layers. This representation was then provided as input to a deep learning framework based on a CNN architecture composed of three convolutional layers with 64, 32, and 16 filters (3 × 3 kernels), followed by fully connected layers with 16 and 8 units. ReLU activations were applied to all intermediate layers, while a sigmoid activation was employed for the binary classification output. To reduce the risk of overfitting, dropout layers were incorporated after the dense layers, and early stopping was applied during training. The full architecture of LUSC stage classification late fusion was presented in Fig. 4.

#### Model 2 — sDCFE hyper model

**Fig. 5:**
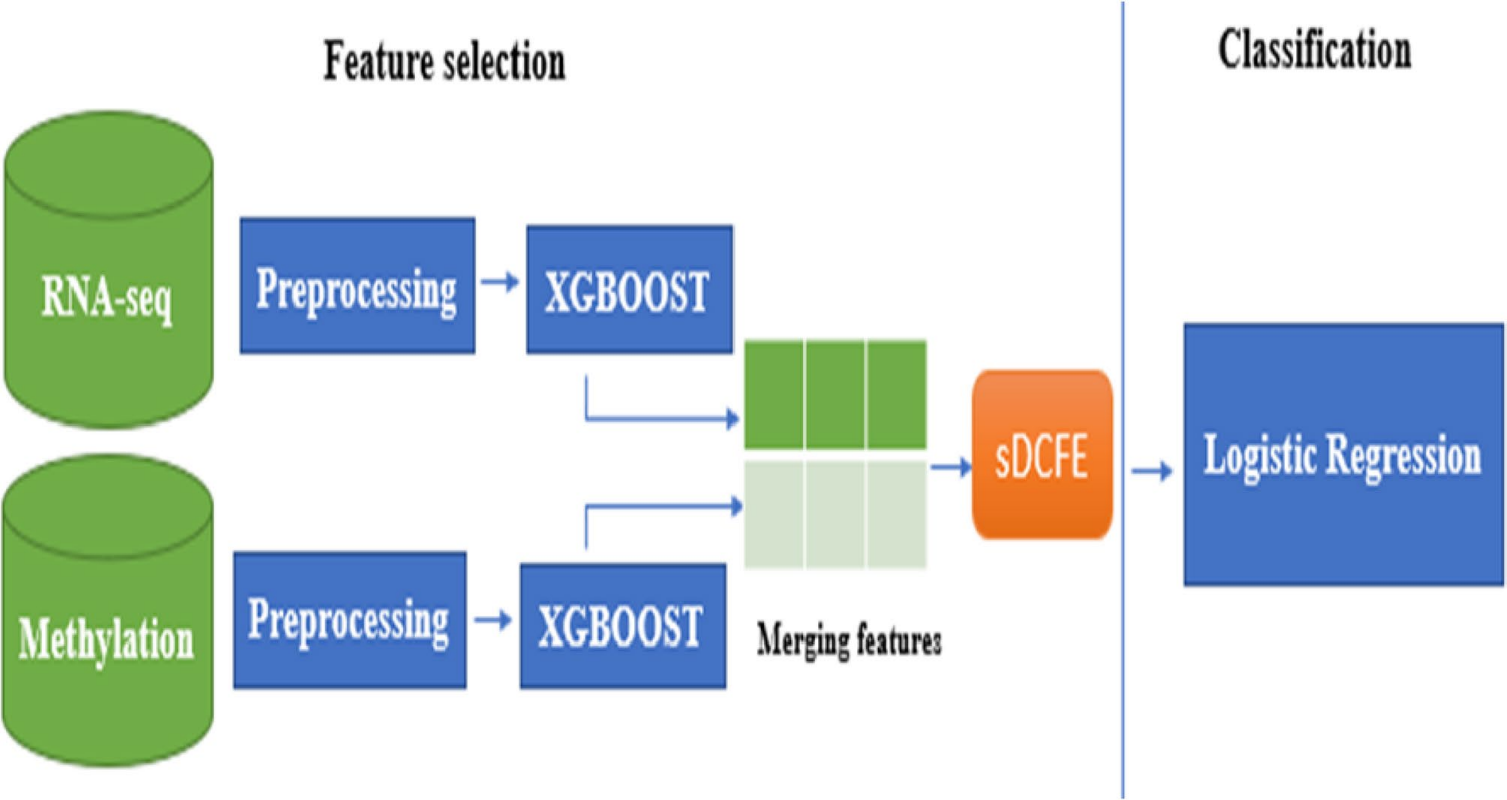
Architecture of LUSC stage classification sDCFE Hyper Model

To enhance stability and interpretability, the sDCFE method was applied to the integrated feature set obtained after XGBoost selection; 921 integrated features. Genes were rescored by sDCFE, and a 50% cutoff was applied, through which the top 461 features were retained. Given the reduced dimensionality, a logistic regression classifier with L2 regularization was employed, as deep learning was not required for this smaller feature space. Figure 5 illustrates the LUSC classification framework, in which the sDCFE-based hyper-model are depicted.

### Functional and biological analyses TCGA Pancancer of the intersected genes

#### Enrichment analysis

Functional enrichment of the intersected genes signature was carried out using the Enrichr tool [33] as implemented in the GSEApy Python package [34] and R clusterProfiler package [35]. The KEGG 2021 Human [36], Reactome 2022 [37], and GO Biological Process 2023 (GO BP) [38] libraries were interrogated to identify significantly overrepresented pathways. Enrichment significance was determined using Fisher’s exact [39] test followed by Benjamini–Hochberg false discovery rate (FDR) correction [40]. Terms with adjusted p-values (FDR) ≤ 0.05 were retained as significant. Results were ordered by Combined Score (log(p) × z-score), and the top 20 enriched terms from each database were depicted using dot plots.

#### Survival analysis

Survival associations for the selected genes were evaluated using Kaplan–Meier (KM) [41] analysis and Cox proportional hazards regression [42]. Patients were stratified into high- and low-expression groups on the basis of median expression values, and survival differences were assessed with the log-rank test. Cox regression models were applied to estimate hazard ratios (HRs) together with 95% confidence intervals (CIs). Both KM curves and Cox regression outputs were generated and reported to determine the prognostic significance of the identified genes.

#### Novelty assessment

The intersected genes obtained from XGBoost and sDCFE (82) were evaluated to determine whether they represented established or novel biomarkers. A systematic screening was conducted against six resources: four curated biomarker/driver databases (COSMIC Cancer Gene Census [43], OncoKB [44], CIViC [45], and ONGene [46]) and two cancer association resources (Human Protein Atlas – Pathology Atlas [47] and DisGeNET [48]).

##### Biomarker classification framework

To further systematize the novelty assessment, a biomarker classification framework was applied. For each intersected gene, presence or absence in a given database was encoded as a binary value (1 = present, 0 = absent). This binary encoding was applied to enable systematic comparison across resources.

For each gene, the mean score across the six binary-coded resources was calculated, and classification was assigned based on both the score and database category. Three tiers were defined:

- *Old biomarker*: genes present in two cancer association resources (HPA, DisGeNET) and at least one curated biomarker database (COSMIC, OncoKB, CIViC, ONGene), defined as those with a mean presence score ≥ 0.5 and ≤ 1.
- *Emerging candidate: *genes absent from all four curated databases but present in both cancer association resources (HPA, DisGeNET), defined as those with a mean presence score > 0 and < 0.5.
- *Novel candidate:* genes absent from all six resources, defined as those with a mean presence score = 0.

### Datasets

All datasets employed in this study were obtained from The Cancer Genome Atlas (TCGA) [49], with external validation conducted using the Pancancer Analysis of Whole Genomes (PCAWG) [50] project, which is a large-scale international collaboration under the ICGC (International Cancer Genome Consortium) and TCGA (The Cancer Genome Atlas). Two classification tasks were considered: pancancer classification across ten cancer types, and lung squamous cell carcinoma (TCGA-LUSC) [51] stage classification.

#### Pancancer datasets

The TCGA pancancer cohort consisted of 4,068 RNA-seq tumors samples distributed across ten cancer types: uterine corpus endometrial carcinoma (TCGA-UCEC, 548 samples), thyroid carcinoma (TCGA-THCA, 503), stomach adenocarcinoma (TCGA-STAD, 443), prostate adenocarcinoma (TCGA-PRAD, 499), lung adenocarcinoma (TCGA-LUAD, 522), liver hepatocellular carcinoma (TCGA-LIHC, 377), kidney renal papillary cell carcinoma (TCGA-KIRP, 291), colorectal adenocarcinoma (TCGA-COADREAD, 629), brain lower grade glioma (TCGA-LGG, 515), and bladder urothelial carcinoma (TCGA-BLCA, 412). Each sample was characterized by 20,104 gene expression features, resulting in a total input matrix of approximately 4.8 million data points.

For external validation, an independent PCAWG cohort was employed, which contained 669 tumors samples from the same ten cancer types represented in TCGA. The distribution of PCAWG samples was as follows: UCEC (51), THCA (48), STAD (30), PRAD (20), LUAD (90), LIHC (119), KIRP (185), COADREAD (55), LGG (48), and BLCA (23). PCAWG was chosen as the external dataset because it includes the same cancer types as TCGA, thereby allowing the generalizability of the classification framework to be directly evaluated.

#### LUSC datasets

For the TCGA-LUSC stage classification task, two omics datasets were analyzed. The RNA-seq dataset comprised 406 samples **(**244 Stage I and 162 Stage II**)** with 20,104 gene expression features per sample. The DNA methylation dataset comprised 307 samples **(**172 Stage I and 135 Stage II) with 20,104 CpG features per sample. For multiomics integration, only patients for whom both RNA-seq and methylation profiles were available were retained, yielding a matched dataset of 307 samples (172 Stage I, 135 Stage II).

All TCGA datasets were partitioned into training and testing **sets** using a fixed 80%/20% split, and this division was consistently applied across all experiments to ensure comparability. The PCAWG dataset was used exclusively as an independent unseen test cohort for the pancancer classification task.

## Results

### Performance metrics

The performance evaluation phase employed accuracy as the primary metric for all the experiments. For the experiment yielding the best results, additional metrics were used, including precision (as defined in (1)), recall (as outlined in (2)), F1 score (as described in (3)), and accuracy, as indicated in (4)) [52]. These metrics provide a comprehensive assessment of the model’s effectiveness in classifying cancer stages. By evaluating precision and recall, we were able to understand the model’s performance in terms of both its ability to correctly identify positive cases and its sensitivity to detecting all relevant instances. The F1 score, which balances precision and recall, offered further insight into the overall balance between these two critical factors, ensuring that the model’s predictions were both accurate and reliable.

3 $$\:\mathrm{P}\mathrm{r}\mathrm{e}\mathrm{c}\mathrm{i}\mathrm{s}\mathrm{i}\mathrm{o}\mathrm{n}=\frac{\mathrm{t}\mathrm{r}\mathrm{u}\mathrm{e}\:\mathrm{p}\mathrm{o}\mathrm{s}\mathrm{i}\mathrm{t}\mathrm{i}\mathrm{v}\mathrm{e}\mathrm{s}}{\mathrm{t}\mathrm{r}\mathrm{u}\mathrm{e}\:\mathrm{p}\mathrm{o}\mathrm{s}\mathrm{i}\mathrm{t}\mathrm{i}\mathrm{v}\mathrm{e}\mathrm{s}+\mathrm{f}\mathrm{a}\mathrm{l}\mathrm{s}\mathrm{e}\:\mathrm{p}\mathrm{o}\mathrm{s}\mathrm{i}\mathrm{t}\mathrm{i}\mathrm{v}\mathrm{e}\mathrm{s}}$$

4 $$\:\mathrm{R}\mathrm{e}\mathrm{c}\mathrm{a}\mathrm{l}\mathrm{l}=\:\frac{\mathrm{t}\mathrm{r}\mathrm{u}\mathrm{e}\:\mathrm{p}\mathrm{o}\mathrm{s}\mathrm{i}\mathrm{t}\mathrm{i}\mathrm{v}\mathrm{e}\mathrm{s}}{\mathrm{t}\mathrm{r}\mathrm{u}\mathrm{e}\:\mathrm{p}\mathrm{o}\mathrm{s}\mathrm{i}\mathrm{t}\mathrm{i}\mathrm{v}\mathrm{e}\mathrm{s}+\mathrm{f}\mathrm{a}\mathrm{l}\mathrm{s}\mathrm{e}\:\mathrm{n}\mathrm{e}\mathrm{g}\mathrm{a}\mathrm{t}\mathrm{i}\mathrm{v}\mathrm{e}\mathrm{s}}$$

5 $$\:\mathrm{F}1-\mathrm{s}\mathrm{c}\mathrm{o}\mathrm{r}\mathrm{e}=2\times\:\frac{\mathrm{p}\mathrm{r}\mathrm{e}\mathrm{c}\mathrm{i}\mathrm{s}\mathrm{i}\mathrm{o}\mathrm{n}\times\:\mathrm{r}\mathrm{e}\mathrm{c}\mathrm{a}\mathrm{l}\mathrm{l}}{\mathrm{p}\mathrm{r}\mathrm{e}\mathrm{s}\mathrm{i}\mathrm{s}\mathrm{i}\mathrm{o}\mathrm{n}+\mathrm{r}\mathrm{e}\mathrm{c}\mathrm{a}\mathrm{l}\mathrm{l}}$$

6 $$\:\mathrm{Accuracy}=\frac{\mathrm{t}\mathrm{h}\mathrm{e}\:\mathrm{n}\mathrm{u}\mathrm{m}\mathrm{b}\mathrm{e}\mathrm{r}\:\mathrm{f}\:\mathrm{c}\mathrm{o}\mathrm{r}\mathrm{r}\mathrm{e}\mathrm{c}\mathrm{t}\:\mathrm{p}\mathrm{r}\mathrm{e}\mathrm{d}\mathrm{i}\mathrm{c}\mathrm{t}\mathrm{i}\mathrm{o}\mathrm{n}\mathrm{s}}{\mathrm{t}\mathrm{o}\mathrm{t}\mathrm{a}\mathrm{l}\:\mathrm{n}\mathrm{u}\mathrm{m}\mathrm{e}\mathrm{r}\:\mathrm{o}\mathrm{f}\:\mathrm{p}\mathrm{r}\mathrm{e}\mathrm{d}\mathrm{i}\mathrm{c}\mathrm{t}\mathrm{i}\mathrm{o}\mathrm{n}\mathrm{s}}\times\:100\%$$

To provide a rigorous and balanced evaluation, multiple performance metrics were employed beyond simple accuracy.

For pancancer classification (10-class task), the following metrics were reported: Accuracy (Acc), Balanced Accuracy (BalAcc), Macro-F1, Matthews Correlation Coefficient (MCC), Macro AUC (OvR), and Expected Calibration Error (Macro-ECE**)**. In addition, ROC curves were plotted (one-vs-rest to illustrate the trade-offs between sensitivity and specificity, and to enable model comparison independent of threshold choice or class imbalance, Calibration plots were generated to assess probability reliability by evaluating how well predicted probabilities aligned with observed outcomes. In addition, a row-normalized confusion matrix was reported, in which the predicted labels were compared with the true labels on a per-class basis, providing a fair and comprehensive picture of the classifier’s performance independent of class sample sizes. Macro-averaging was applied across classes to ensure equal weighting.

For LUSC stage classification (binary task**)**, the reported metrics included Accuracy, F1-score, and AUC. ROC curves and confusion matrices were employed to visualize discriminative ability and probability calibration, while Precision–Recall curves were used to assess classifier performance on imbalanced datasets by plotting precision against recall across varying thresholds. All metrics were computed on the fixed TCGA training/testing split, with external validation performed on the PCAWG cohort to assess generalizability.

### Pancancer classification experiment

#### TCGA cohort

Pancancer classification was conducted using the TCGA RNA-seq cohort of 4,068 samples across ten cancer types. Feature selection was applied separately using XGBoost and sDCFE, yielding 937 and 350 features, respectively. After removing redundancy, a combined set of 1,205 nonredundant genes was retained as input to the deep learning classifier. For XGBoost, features with positive importance scores (importance > 0) were included, while for sDCFE, the top 350 features were selected through cross-validation, where both feature stability and classification accuracy were observed to plateau beyond this threshold, with minimal gains from including additional genes.

The hybrid model (XGBoost + sDCFE + DL) achieved near-perfect performance on the TCGA test split, with an accuracy of 99.3%, balanced accuracy of 98.8%, macro-F1 of 99.0%, MCC of 99.3%, macro-AUC of 1.0, and an expected calibration error (ECE) of 0.002 (Table 1).

**Fig. 6:**
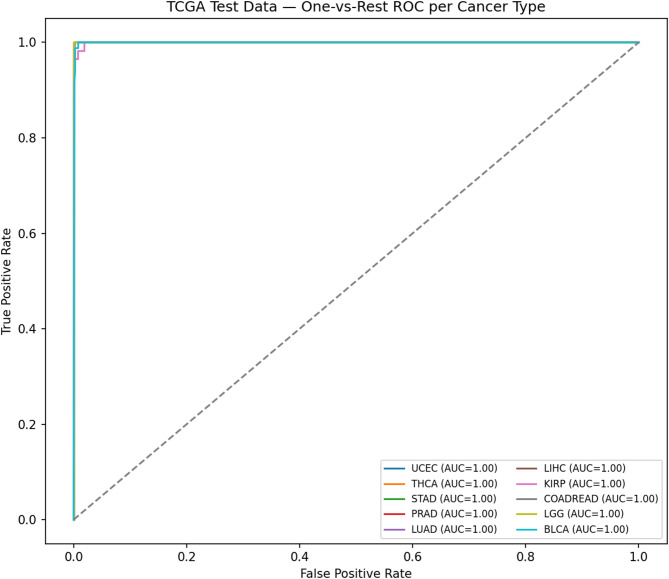
ROC curves for TCGA Pancancer classification

**Fig. 7:**
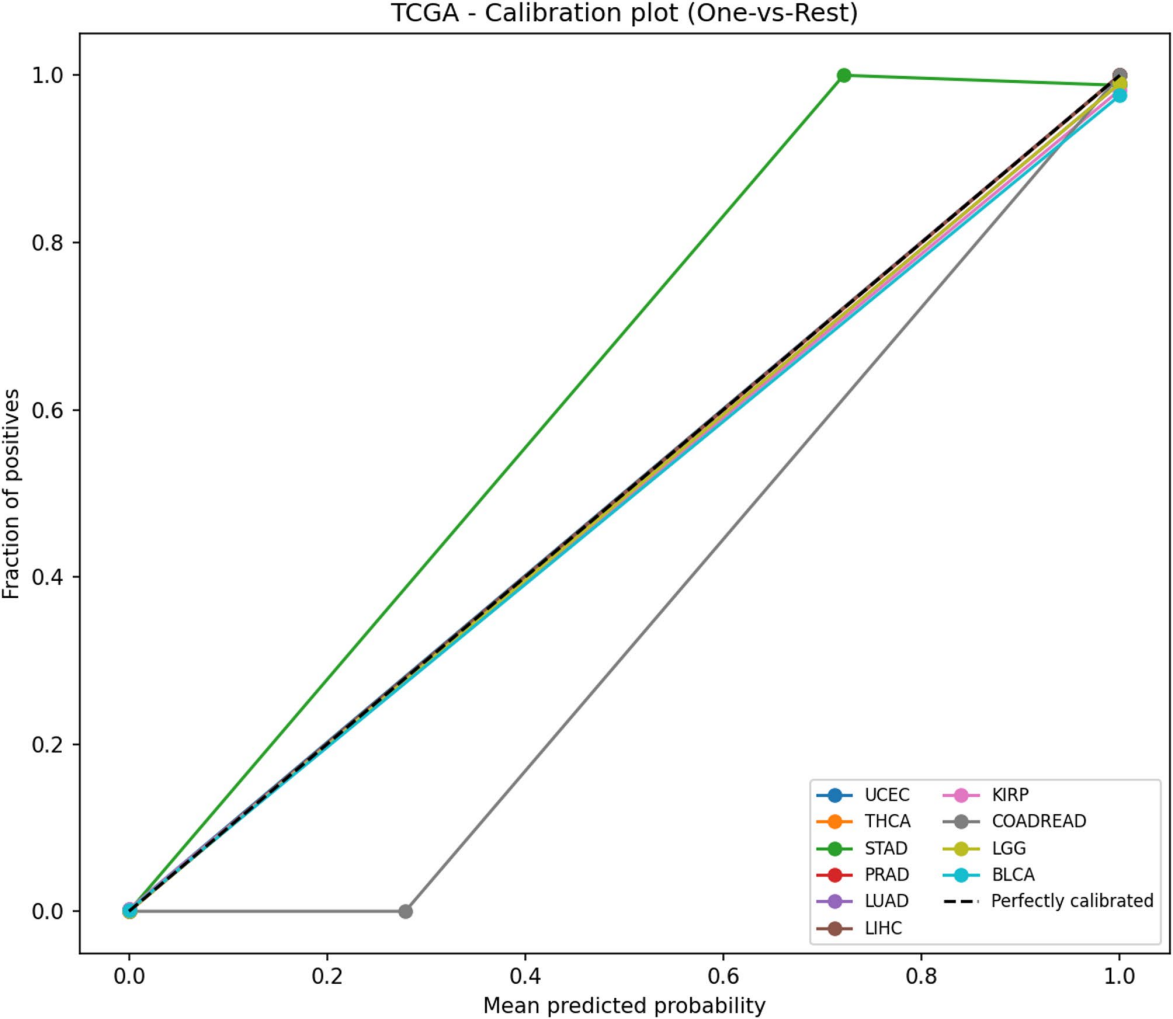
Calibration plot for TCGA Pancancer classification

**Fig. 8:**
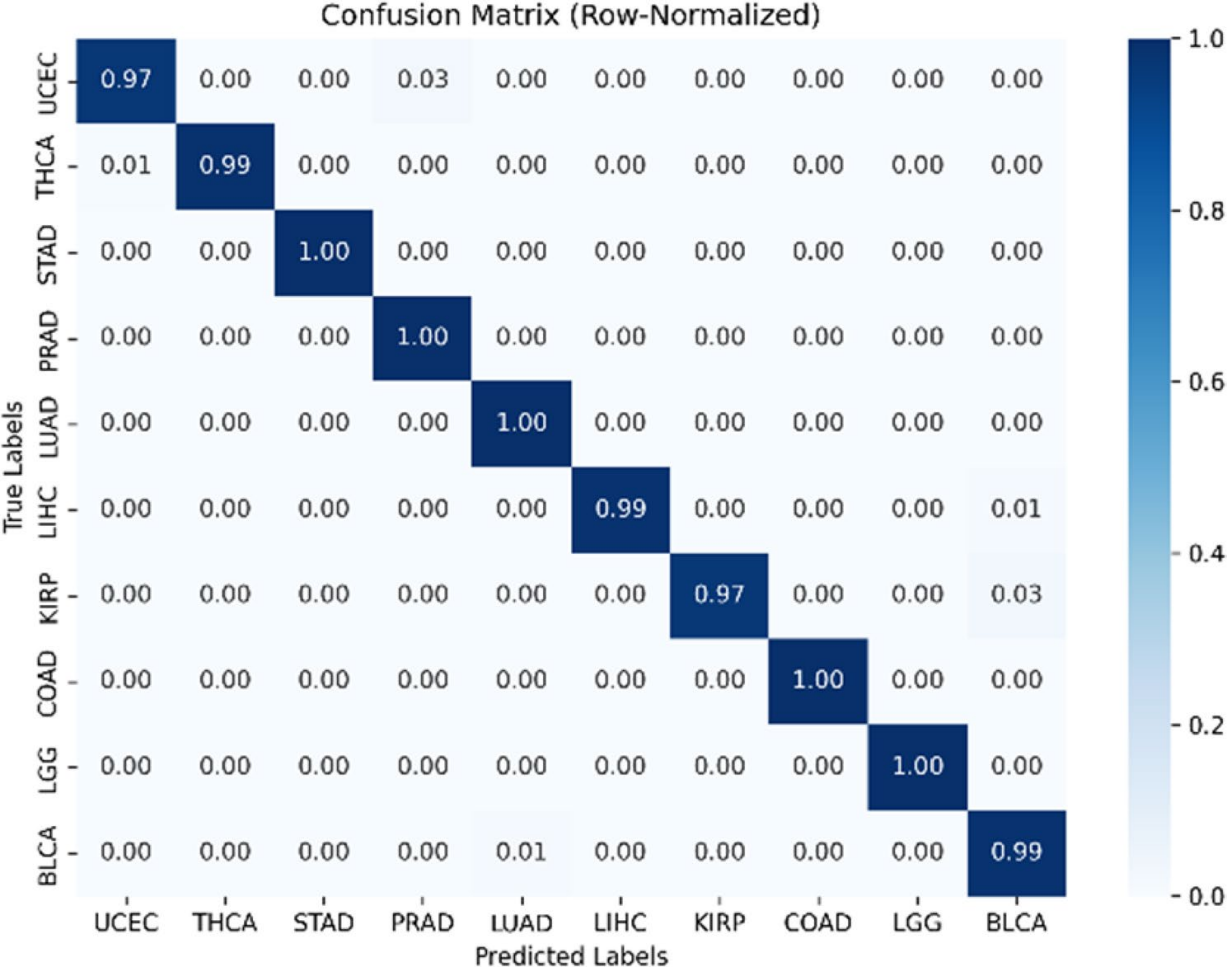
Row-normalized confusion matrix for TCGA Pancancer classification

**Fig. 9:**
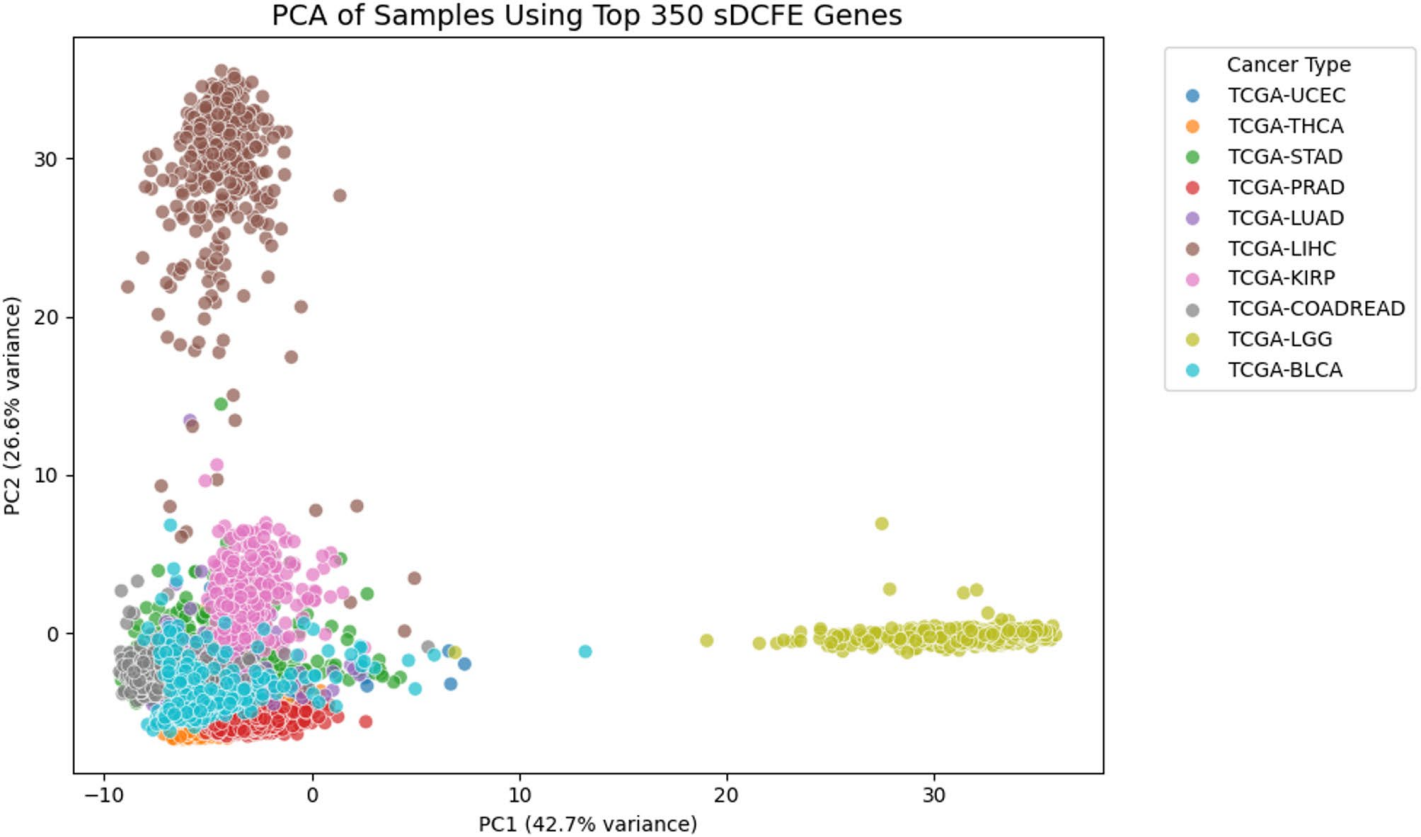
PCA plot of 4068 samples using the top 350 sDCFE-selected genes, colored by cancer type

As illustrated in Fig. 6, ROC curves demonstrated strong discriminative performance across all ten cancer types using a one-vs-rest strategy. Probability estimates were well calibrated, as shown in the calibration plot Fig. 7, where predicted probabilities closely aligned with observed outcomes. Per-class prediction results were further summarized by the row-normalized confusion matrix Fig. 8, in which only limited misclassification between cancer types was displayed. A PCA plot generated using the top 350 sDCFE-selected genes in Fig. 9 clearly shows the separation of samples by cancer type. This visualization confirms the discriminative power of the selected features and supports the utility of sDCFE for pancancer classification tasks. Detailed precision, recall, and F1-scores for each cancer type are provided in Supplementary Table S1. Together, these results underscore the robustness of the framework and its potential to capture both global and cancer-specific signals. Importantly, the integration of sDCFE with deep learning highlights its capacity to enhance biomarker discovery and improve downstream clinical translation.

#### PCAWG cohort external validation

To further evaluate generalizability, the hybrid XGBoost + sDCFE + DL framework was tested on the independent PCAWG cohort, which contained 669 RNA-seq tumors samples from the same ten cancer types represented in TCGA. A total of 1,205 non-redundant features derived from the TCGA training pipeline were applied without retraining. Of these 300 features were absent in the validation dataset, their values were imputed as zero to maintain feature alignment, which partly explains the observed accuracy drop (from 99.3% to 94%). On this unseen dataset, the model achieved an accuracy of 94%, balanced accuracy of 92%, macro-F1 of 91.2%, MCC of 92.9%, macro-AUC of 99.7%, and an expected calibration error (ECE) of 0.012 (Table 1).

**Fig. 10:**
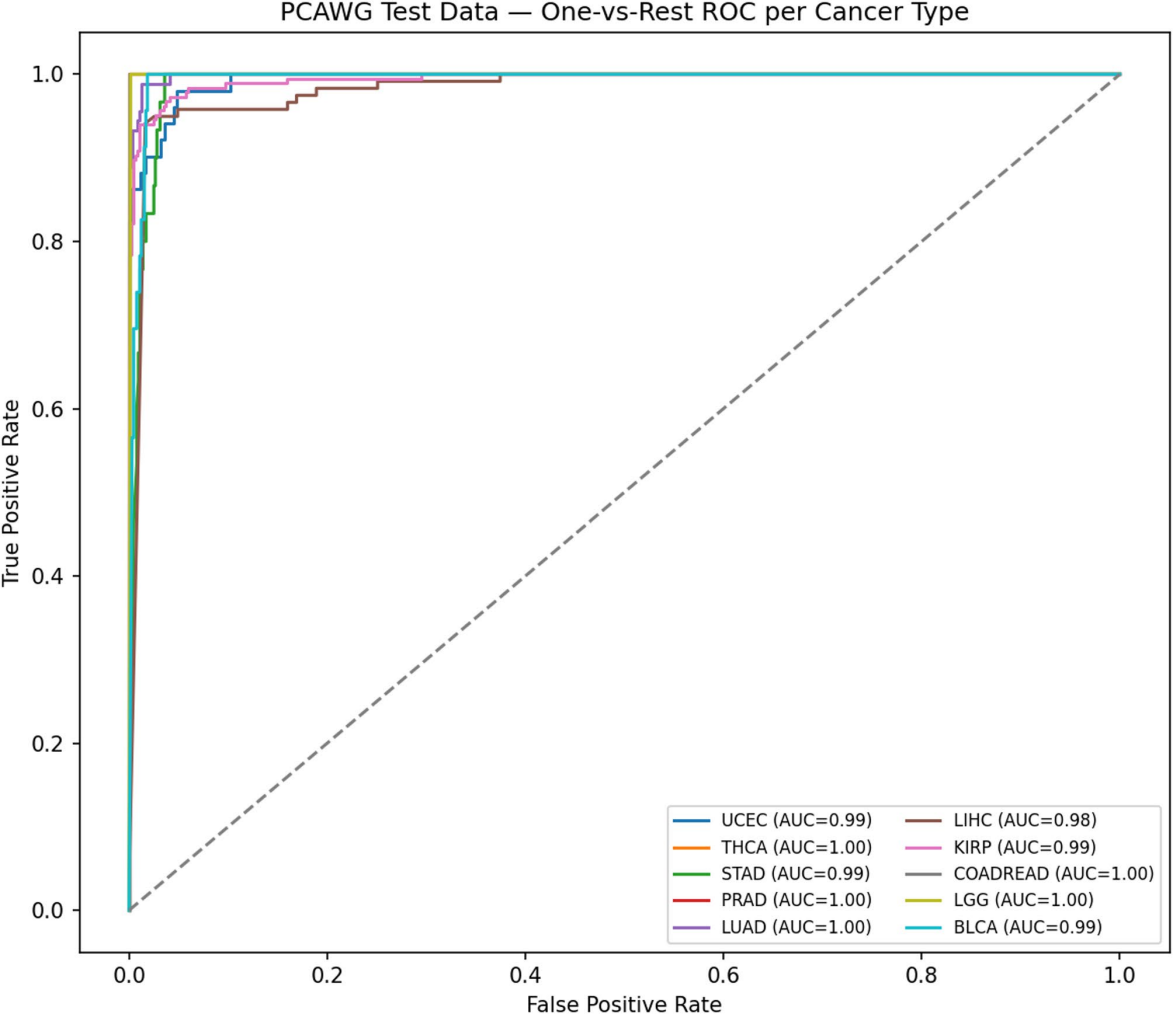
ROC curves for PCAWG Pancancer test

**Fig. 11:**
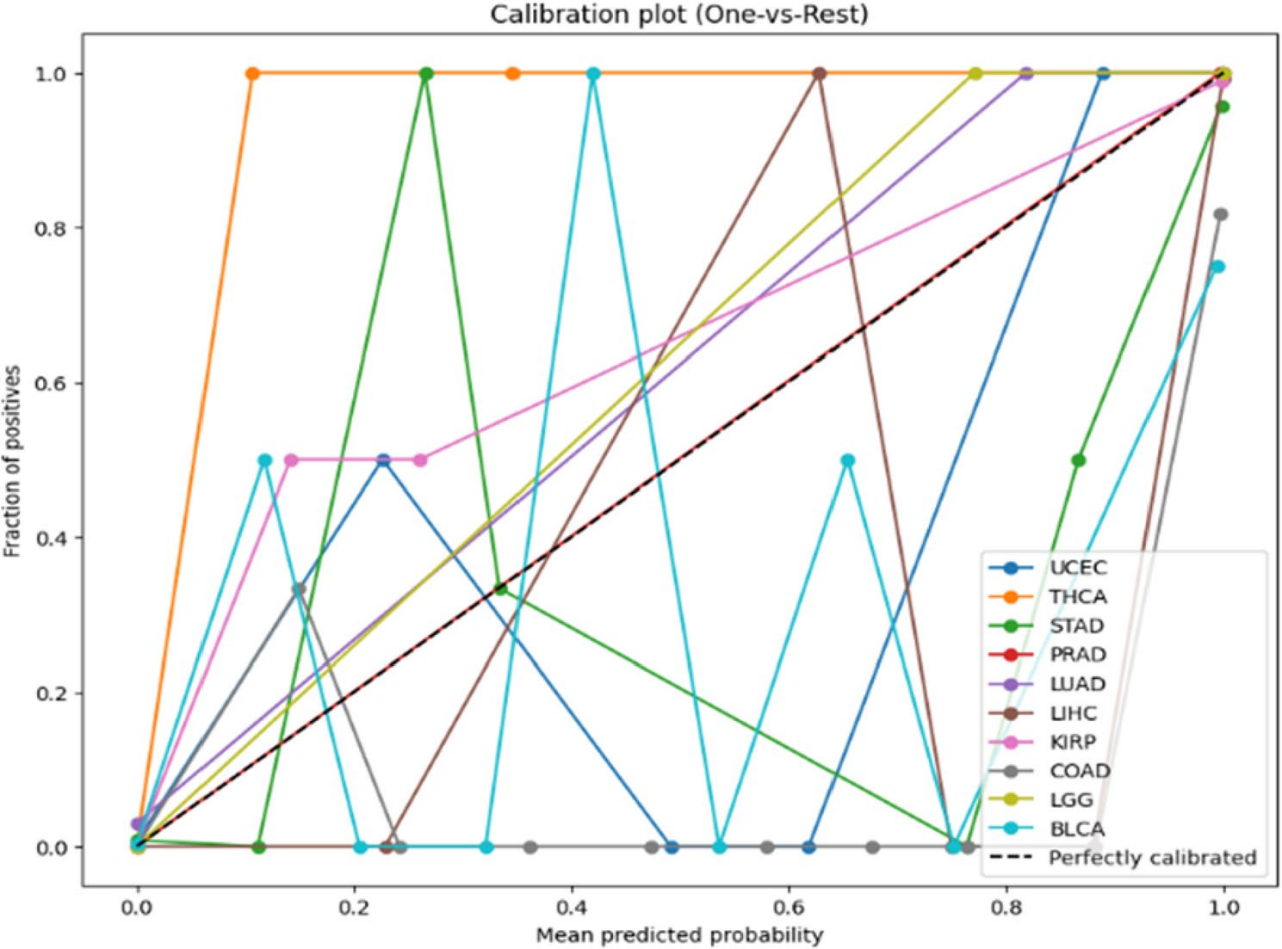
Calibration plot for PCAWG Pancancer test

**Fig. 12:**
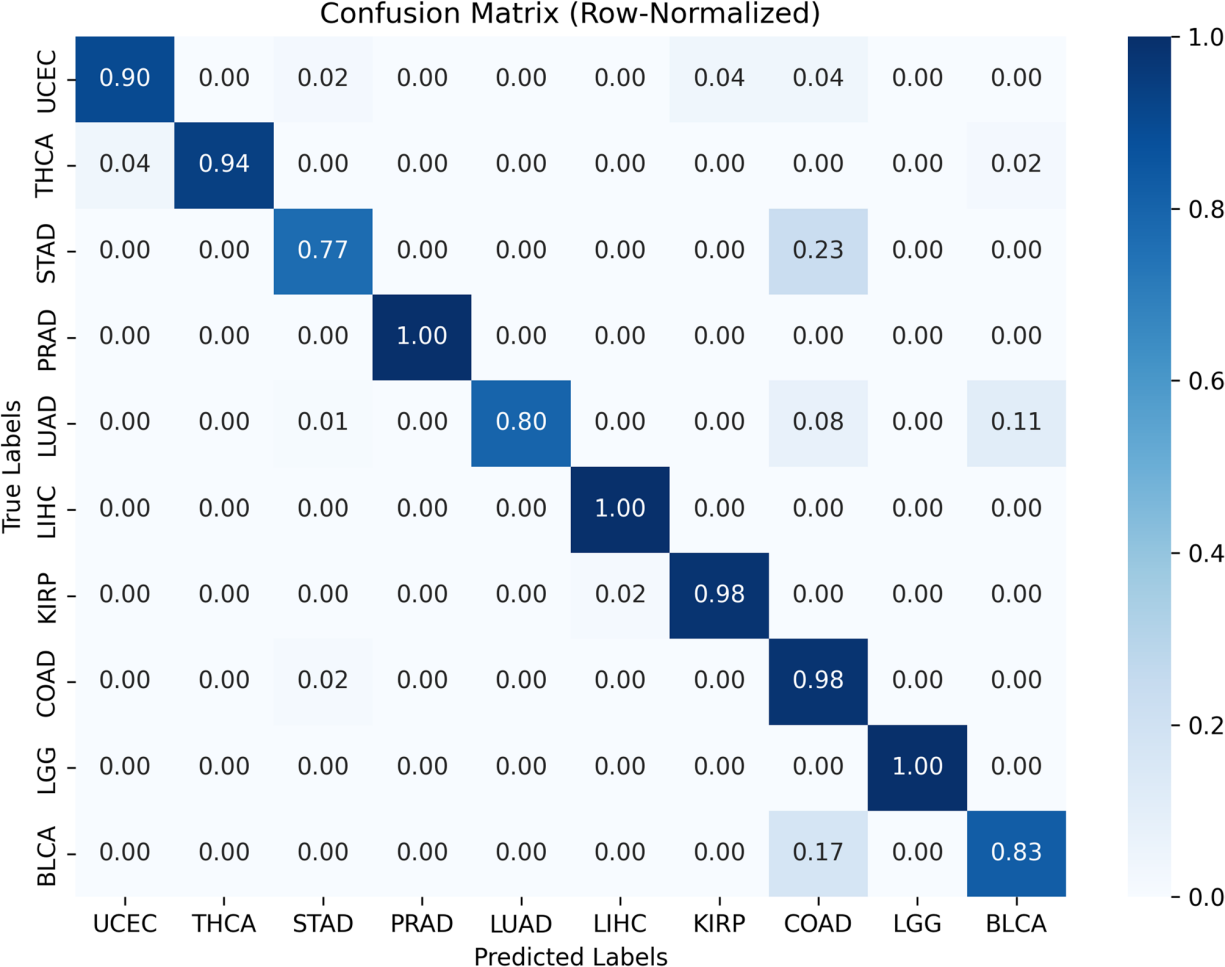
Row-normalized confusion matrix for PCAWG pancancer classification

As illustrated in Fig. 10, ROC curves generated on the PCAWG cohort confirmed strong discriminative performance across the ten cancer types. The calibration plot Fig. 11 indicated well-aligned probability estimates, and the row-normalized confusion matrix Fig. 12 summarized per-class predictions, showing limited misclassification. Detailed per-class classification metrics for PCAWG are provided in Supplementary Table S1.

Overall, accurate and well-calibrated predictions for pancancer classification across both internal and external cohorts were achieved by the hybrid XGBoost + sDCFE + DL model. These findings demonstrate the robustness of the framework when applied to independent datasets. Importantly, the results highlight the ability of sDCFE to capture transferable biological signals rather than dataset-specific artifacts. This strengthens confidence in the model’s potential for clinical application and cross-cohort reproducibility.

#### Baseline classifier evaluation

To benchmark the proposed hybrid framework, several baseline models were evaluated, including XGBoost alone, sDCFE alone, Logistic Regression, SVM, and a combined sDCFE + XGBoost model. All baselines were trained and tested on the same TCGA pancancer cohort comprising 4,068 RNA-seq samples to ensure comparability. The full input space consisted of 20,104 features per sample. From this, sDCFE selected 350 features based on the cross-validation–derived cutoff, while XGBoost identified 937 informative features, which were then provided as inputs to the respective classifiers.

The results are summarized in Table 1. XGBoost alone achieved strong predictive performance with an accuracy of 99.1% and MCC of 0.992, while sDCFE alone yielded slightly lower performance (accuracy = 96.6%, MCC = 0.962), reflecting its role as a feature selection–driven method rather than a high-capacity classifier. Logistic Regression and SVM, when applied to the sDCFE-selected features, demonstrated robust performance (accuracy = 99.2% and 98.7%, respectively), indicating that sDCFE-derived features are highly discriminative and transferable across classifier families. The combination of sDCFE + XGBoost performed comparably to XGBoost alone, maintaining high accuracy (99.1%) and excellent calibration (ECE = 0.002).

Overall, the comparative baselines confirmed that while XGBoost provides a strong standalone baseline, the integration of sDCFE features enhances stability and interpretability, with consistently high performance observed across different classifier types.

**Table 1:** Performance of baseline models compared to the proposed hybrid framework

Model	Acc	BalAcc	MacroF1	MCC	Macro AUC	ECE
sDCFE (alone)	0.966	0.965	0.960	0.962	0.983	0.012
XGB alone	0.991	0.987	0.990	0.991	1.000	0.002
sDCFE + Logistic	0.992	0.988	0.990	0.992	1.000	0.002
sDCFE + SVM	0.987	0.989	0.989	0.989	1.000	0.005
sDCFE + XGB	0.992	0.989	0.989	0.992	1.000	0.002
Proposed model TCGA	0.993	0.988	0.99	0.993	1.000	0.002
Proposed model PCAWG	0.94	0.920	0.912	0.929	0.997	0.012

#### Intersected genes analysis

**Fig. 13:**
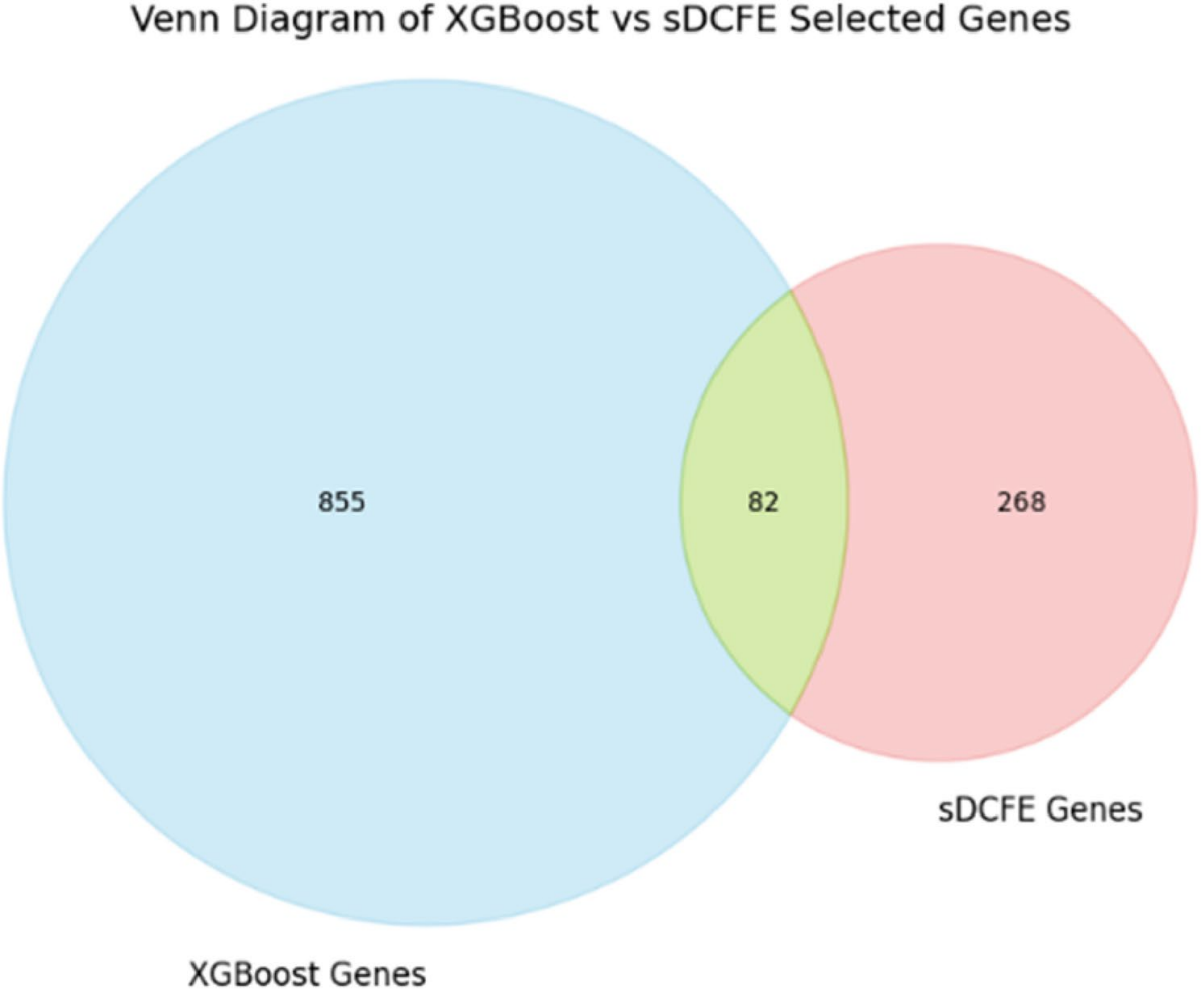
Venn diagram showing the intersection of the top 350 genes selected by XGBoost and sDCFE, highlighting 82 shared genes

The intersection between genes selected by XGBoost and sDCFE was analyzed to identify consistently informative biomarkers. The Venn diagram Fig. 13 illustrates that 82 genes were shared between the two methods. These intersected genes were subsequently subjected to downstream biological validation, including enrichment analysis, survival analysis, and biomarker novelty assessment.

The significance of this overlap lies in the fact that genes independently prioritized by two distinct feature selection approaches are more likely to represent stable and biologically reproducible signals. Such convergence reduces method-specific bias, enhances confidence in their biological relevance, and strengthens their potential utility as robust biomarkers.

### Functional and biological validation TCGA Pancancer of intersected genes

To explore the biological relevance of the 82 intersected genes, functional enrichment, survival analysis, and biomarker novelty assessment were performed.

#### Enrichment analysis

**Fig. 14:**
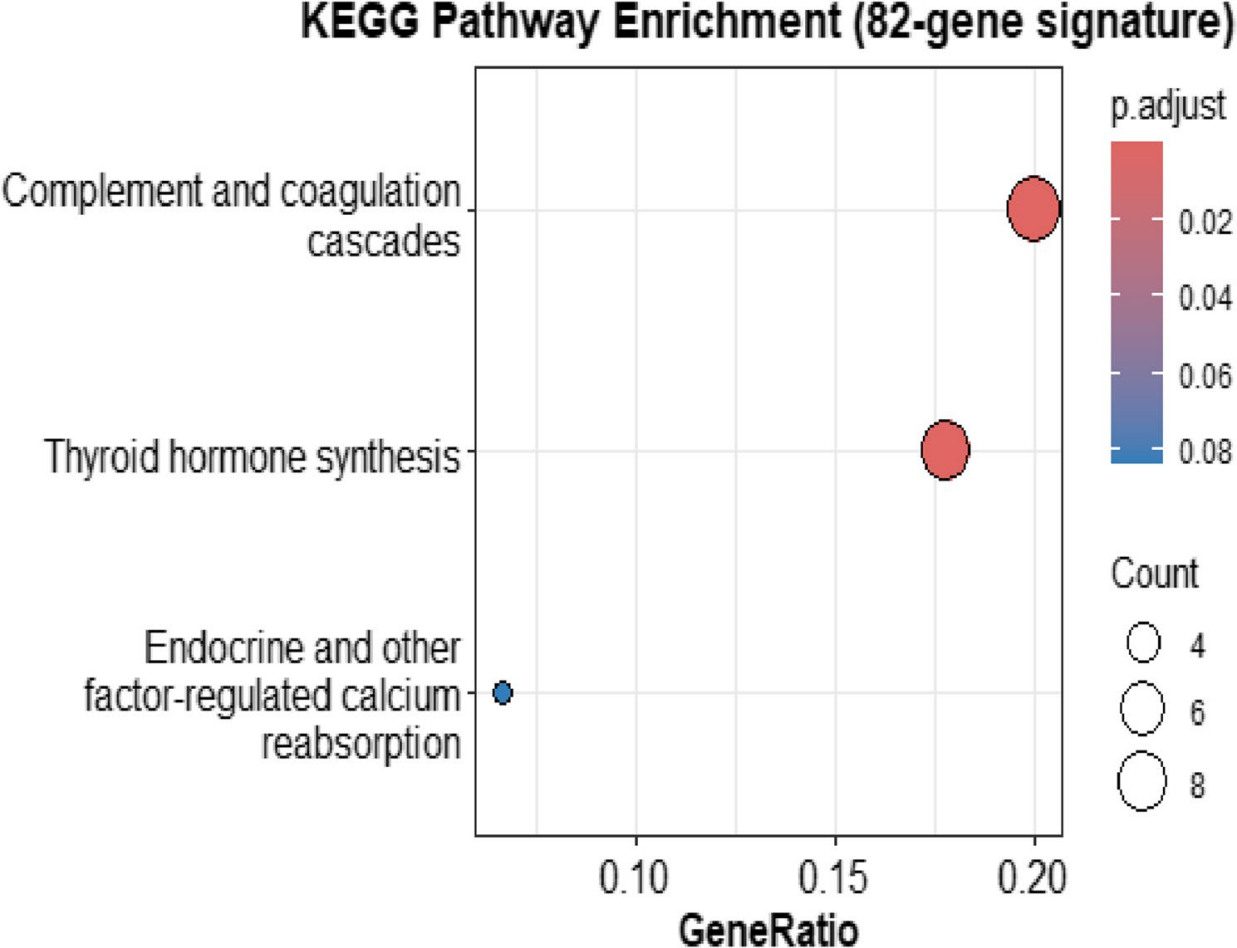
KEGG enrichment of the 82 intersected genes

**Fig. 15:**
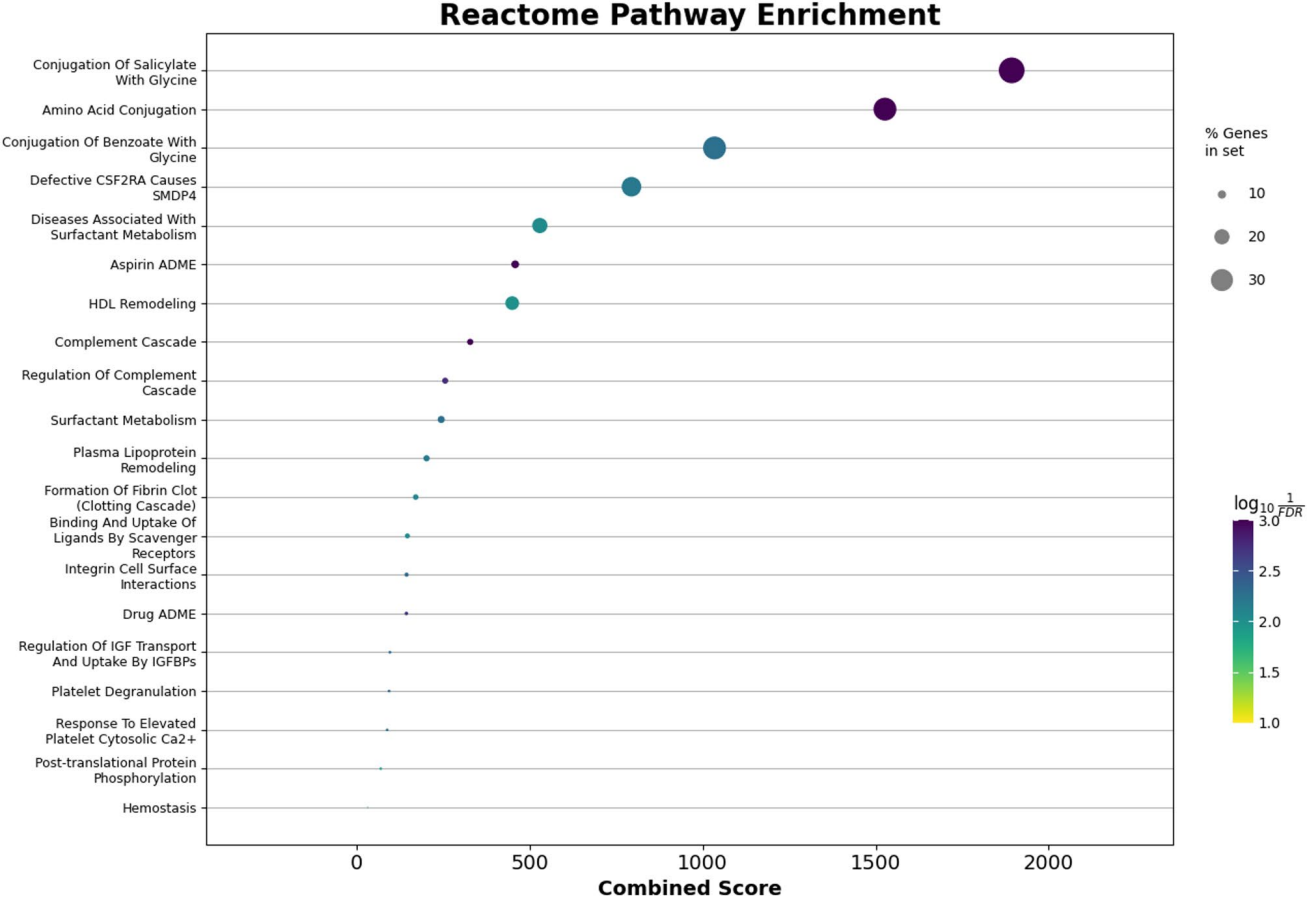
Reactome enrichment of the 82 intersected genes

**Fig. 16:**
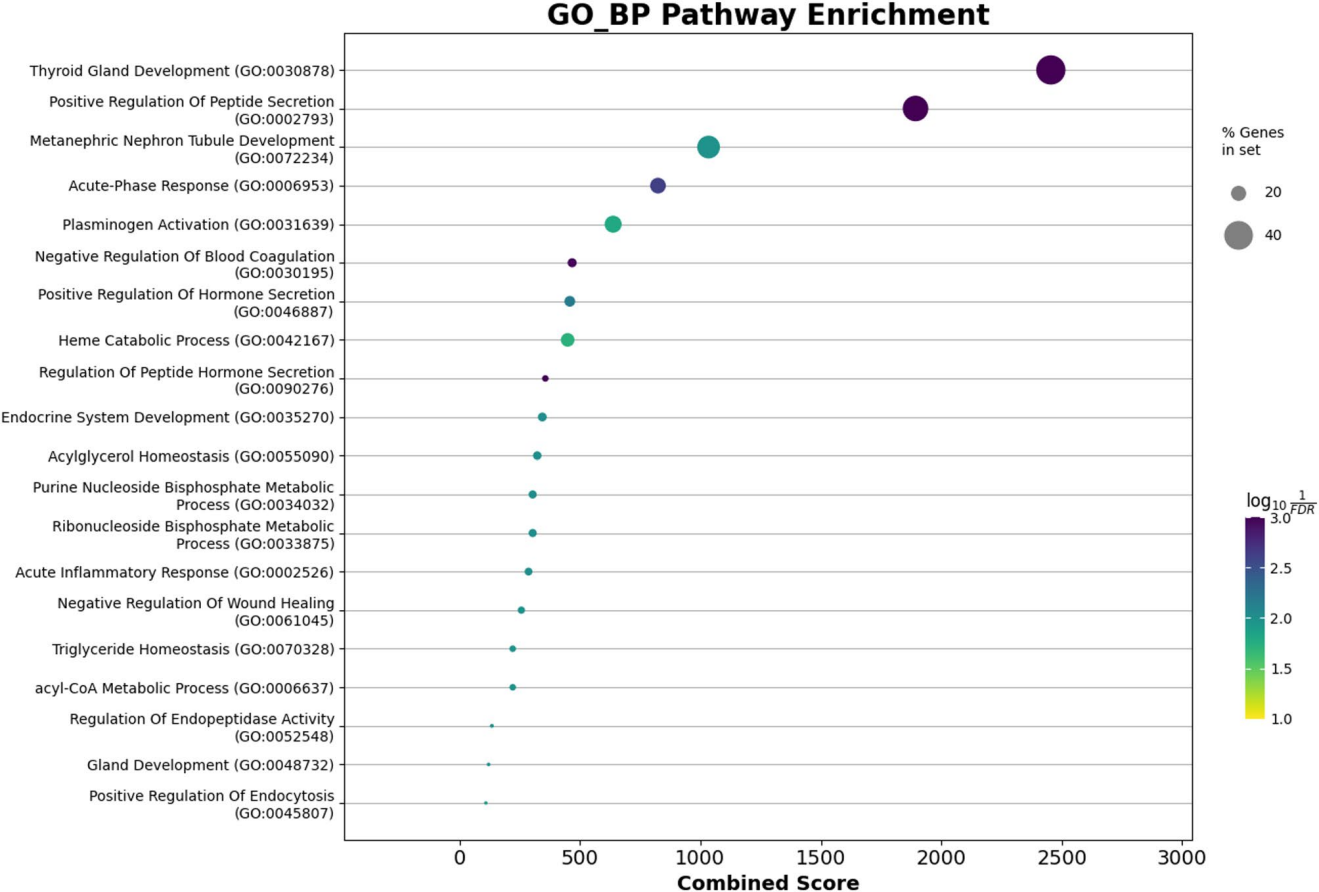
GO BP enrichment of the 82 intersected genes

Functional enrichment analysis was performed using KEGG 2021 Human, Reactome 2022, and GO Biological Process 2023 libraries. The top 20 significantly enriched terms (FDR ≤ 0.05) were identified in each resource and ranked according to combined score. As illustrated in Fig. 14 KEGG, Fig. 15 Reactome, and Fig. 16 GO BP, the KEGG results emphasized pathways central to cancer signalling and cell cycle control, while Reactome analysis highlighted major signalling cascades and metabolic processes. GO BP enrichment revealed a wide range of functional categories, including DNA repair and immune-related mechanisms. Collectively, these findings demonstrate that the overlapping genes selected by sDCFE and XGBoost capture biologically meaningful pathways rather than random associations.

#### Biomarker novelty assessment

Biomarker novelty was assessed by cross-referencing the 82 intersected genes against six curated resources: COSMIC Cancer Gene Census, OncoKB, CIViC, ONGene, the Human Protein Atlas (HPA), and DisGeNET. For each gene, presence or absence in a given database was encoded as binary values (1 = present, 0 = absent), and the mean across resources was calculated. Genes were then systematically classified into three categories:

- *Novel candidate*: mean = 0 (absent from all six resources).
- *Emerging candidate: *0 < mean ≤ 0.5 (present only in cancer association resources, HPA and/or DisGeNET).
- *Established biomarker:* 0.5 < mean ≤ 1.0 (present in at least one curated biomarker/driver database: COSMIC, OncoKB, CIViC, or ONGene).

This analysis revealed 12 established biomarkers, which appeared in HPA, DisGeNET, and at least one curated biomarker/driver database. Sixty-four genes were classified as emerging candidates, present in HPA and DisGeNET but absent from curated biomarker databases. Finally, six genes were identified as completely novel, with no prior annotation in any of the six resources (HFE2, LOC339674, SERINC2, SFTA3, SOX2OT, and ACPP).

This distribution demonstrates that the proposed framework is capable of rediscovering known biomarkers, flagging underexplored candidates, and prioritizing entirely novel genes for future validation. Example subset of intersected biomarkers (*n* = 6) illustrating the three evidence categories Table 2. The complete classification of all 82 intersected genes, including binary presence across six curated resources and per-gene mean scores, is provided in Supplementary Table S2.

**Table 2:** Example subset of intersected biomarkers (*n* = 6) illustrating the three evidence categories

Gene	Mean	Classification category
AMBP	0.3	Emerging candidate
NAPSA	0.3	Emerging candidate
BAIAP2L1	0.6	Established biomarker
FGFR4	0.8	Established biomarker
SFTA3	0	Novel candidate
SOX2OT	0	Novel candidate

#### Survival analysis

To evaluate the prognostic potential of the identified biomarkers, survival analysis was performed using Kaplan–Meier (KM) curves and Cox proportional hazards regression across the ten TCGA cancer cohorts. Hazard ratios (HRs), confidence intervals, and p-values were computed for each of the six candidate genes (SOX2OT, ACPP, SFTA3, SERINC2, LOC339674, and HFE2). The complete results are provided in Supplementary Table S3. Several genes demonstrated strong and statistically significant prognostic effects. In TCGA-LUAD, SFTA3 showed a highly protective effect (HR = 0.73, *p* < 1e-06), while ACPP (HR = 0.83, *p* = 0.010) and SOX2OT (KM borderline significant) also stratified patient outcomes. In TCGA-LIHC, LOC339674 was associated with adverse prognosis (HR = 1.27, *p* = 0.003), whereas HFE2 was protective (HR = 0.81, *p* = 0.006). TCGA-KIRP exhibited protective effects for SERINC2 (HR = 0.76, *p* = 0.020) and adverse effects for LOC339674 (HR = 1.51, *p* = 0.001). In TCGA-COADREAD, SOX2OT (HR = 1.24, *p* = 0.015) and LOC339674 (HR = 1.41, *p* < 0.001) emerged as significant adverse markers. Multiple associations were identified in TCGA-LGG, including protective roles for HFE2 (HR = 0.71, *p* < 0.001) and LOC339674 (HR = 0.45, *p* < 1e-20), and risk associations for ACPP (HR = 1.26, *p* = 0.002) and SERINC2 (HR = 1.63, *p* < 1e-07). Finally, in TCGA-BLCA, SERINC2 was protective (HR = 0.83, *p* = 0.008).

**Fig. 17:**
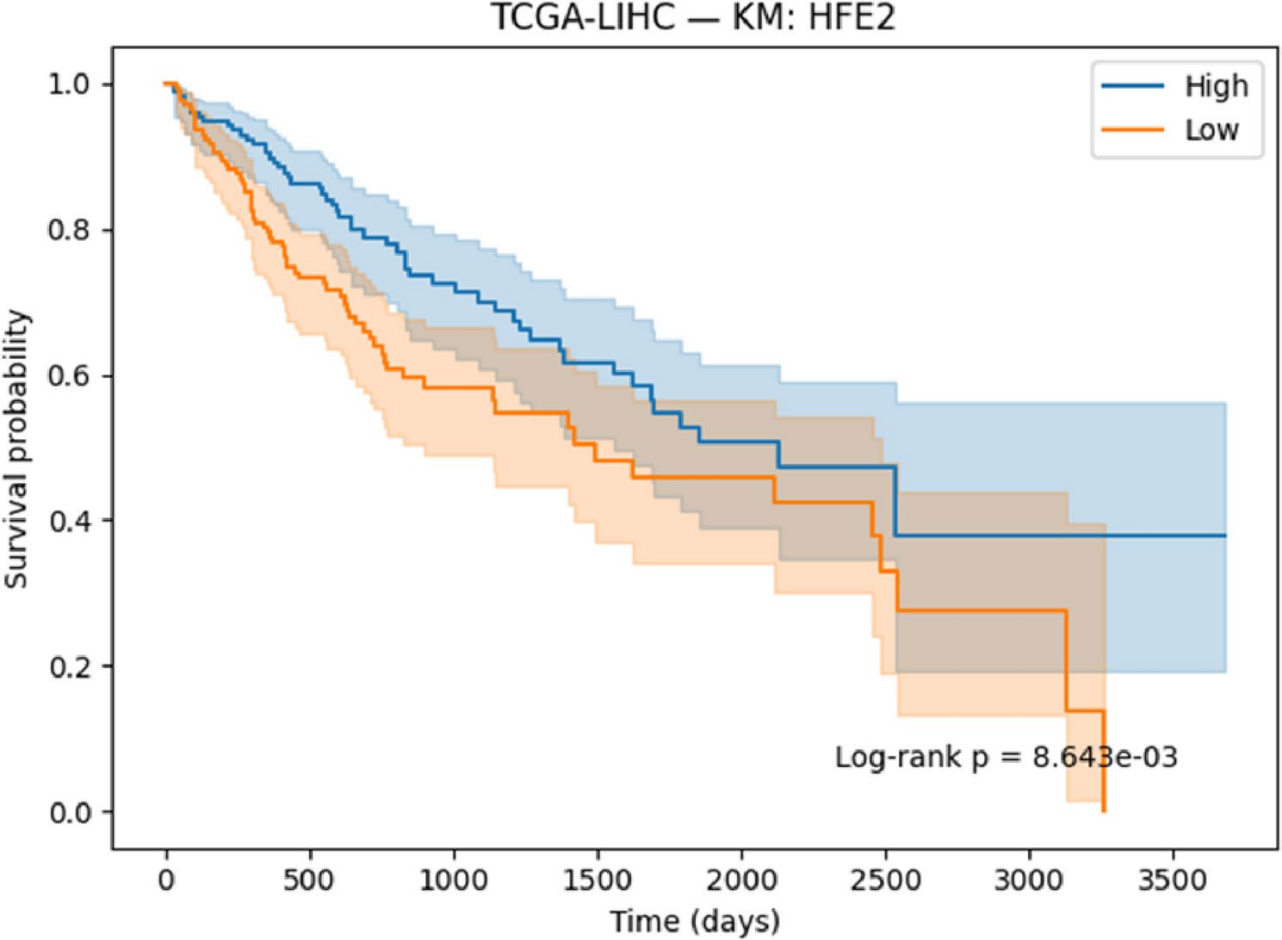
KM survival analysis of HFE2 in LIHC patients, demonstrating a protective effect

**Fig. 18:**
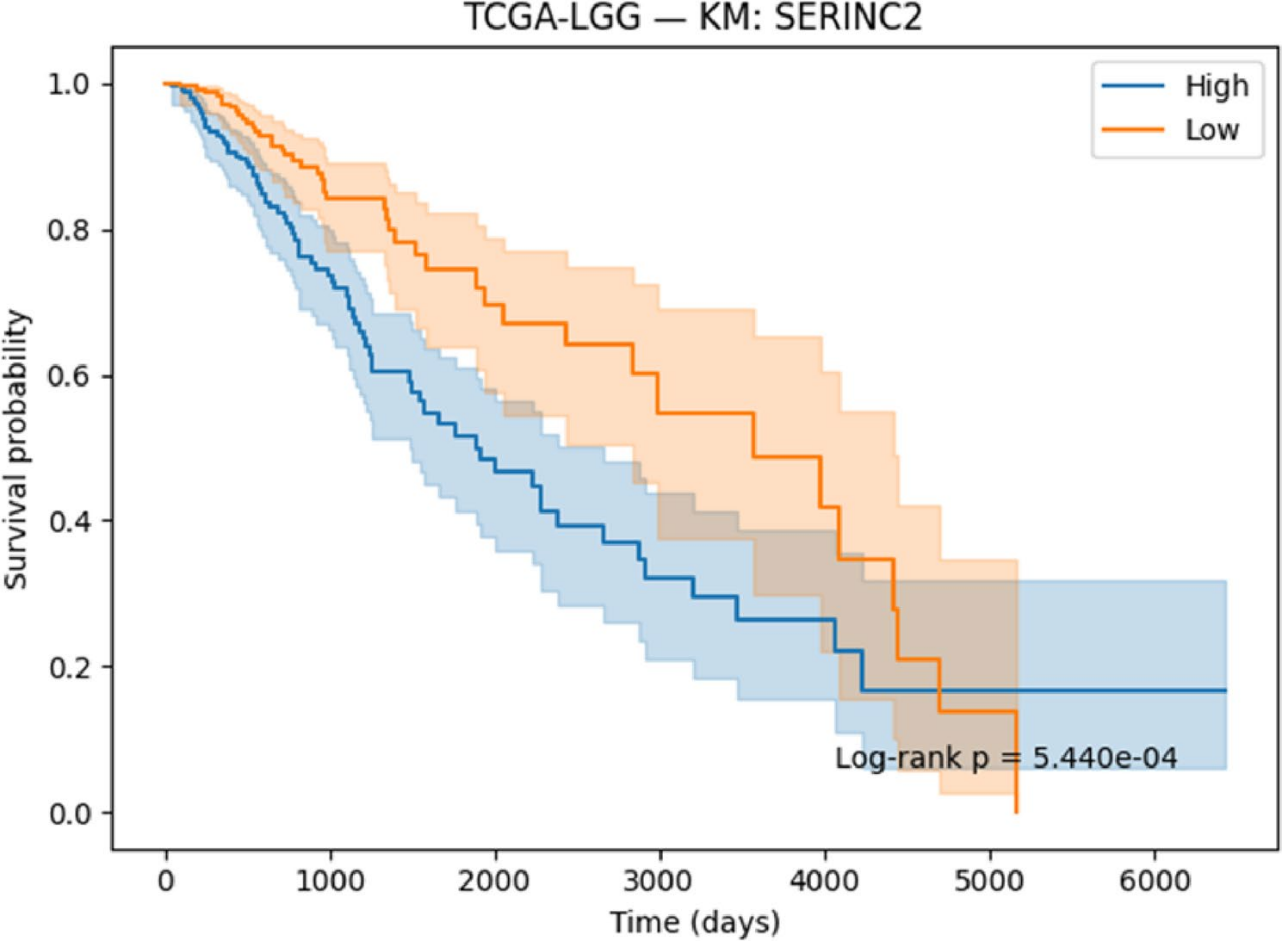
KM survival analysis of SERINC2 in LGG patients, revealing a risk-associated effect (high expression linked to poorer survival

**Fig. 19:**
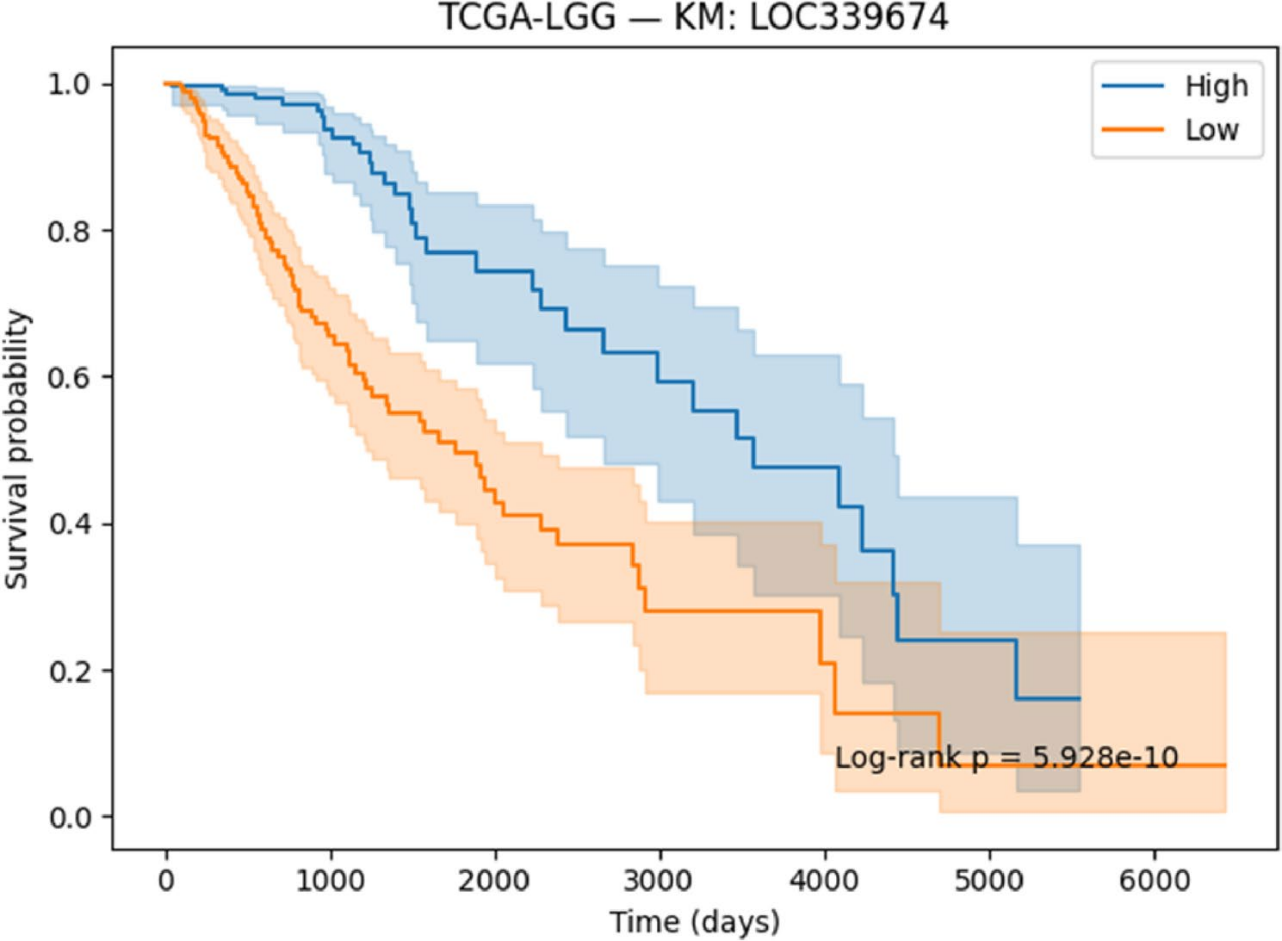
KM survival analysis of LOC339674 in LGG patients, showing a very strong protective association

**Fig. 20:**
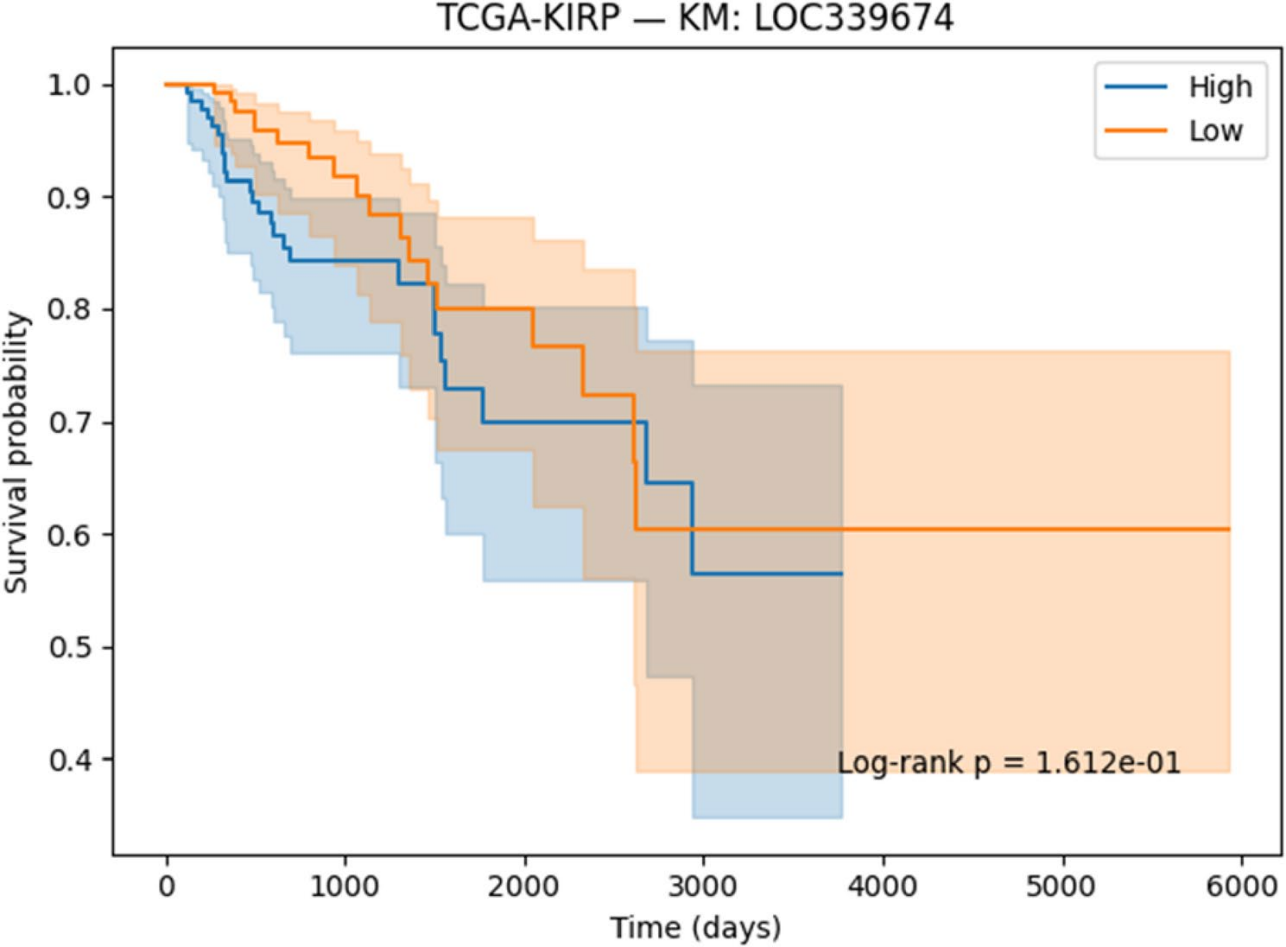
KM survival analysis of LOC339674 in KIRP patients, demonstrating an adverse effect

**Fig. 21:**
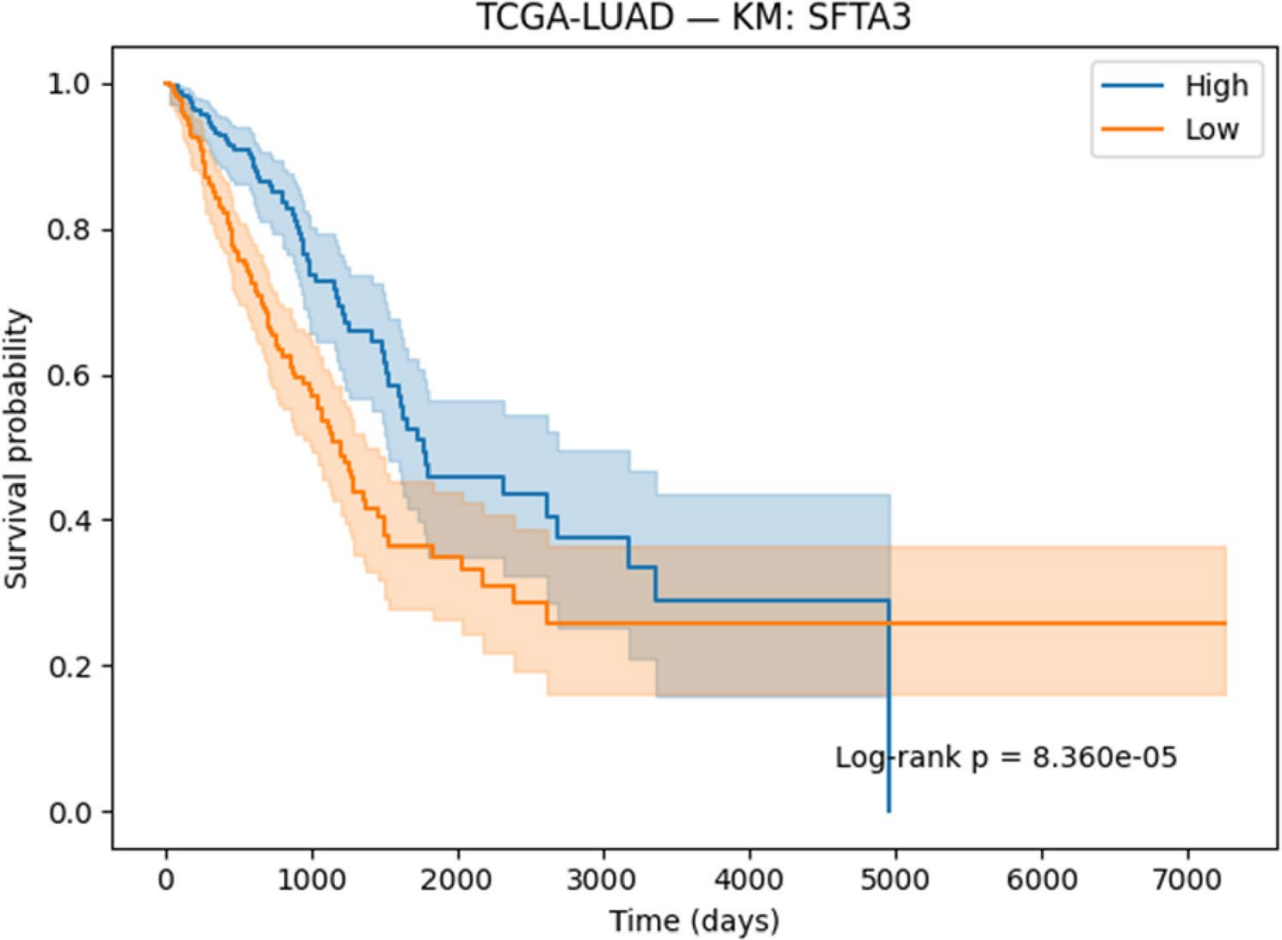
KM survival analysis of SATA3 in LUAD patients, demonstrating an adverse effect

**Fig. 22:**
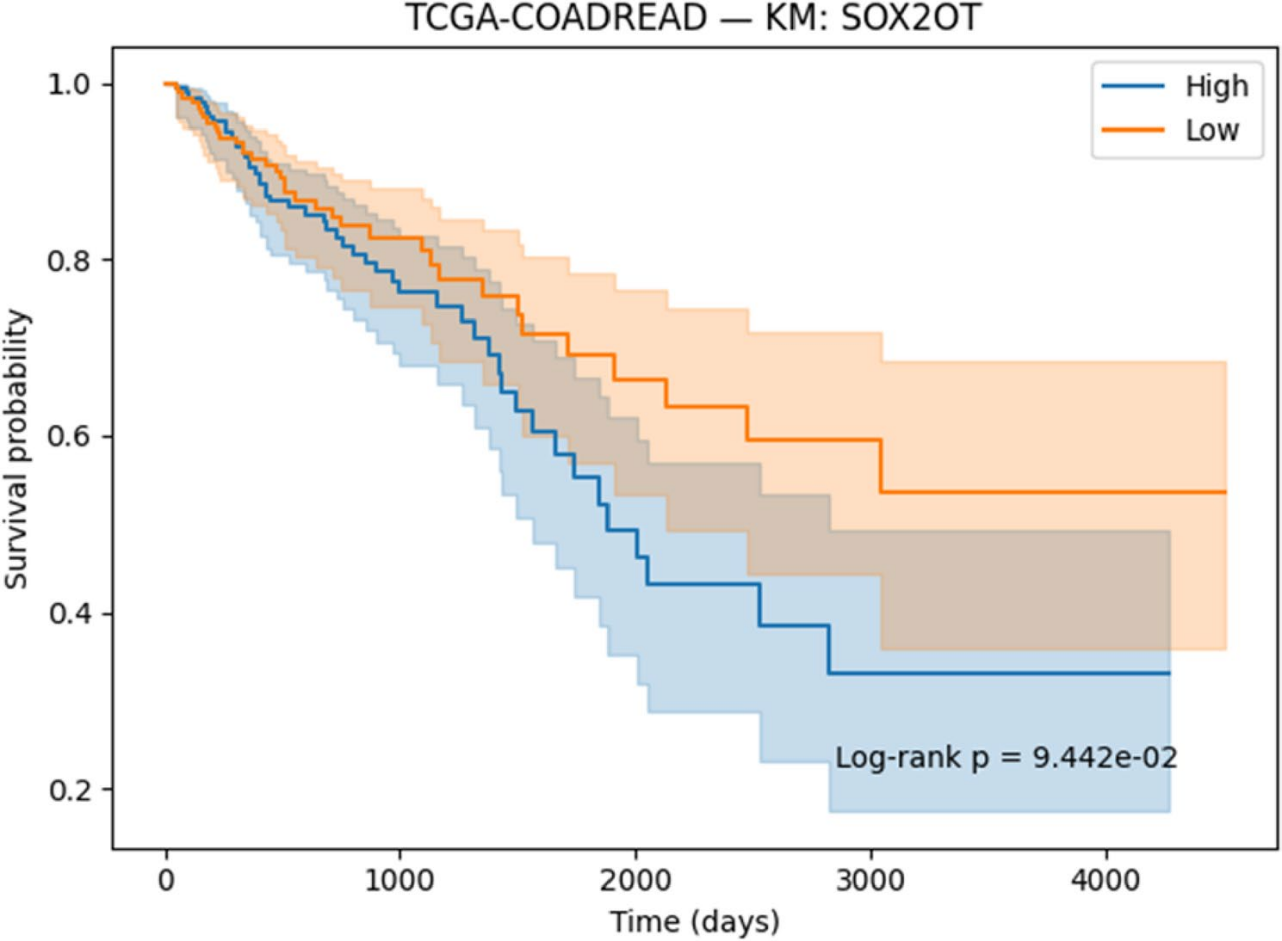
KM survival analysis of SOX2OT in COADREAD patients, demonstrating an adverse effect

Based on significance and effect size, KM plots for the following representative gene–cancer associations are included in the main manuscript Figs. 17, 18, 19, 20, 21 and 22.

Overall, the survival analysis demonstrated that a subset of the 82 intersected genes exhibited significant prognostic associations across multiple cancer types, with both protective and adverse effects observed. These findings highlight the potential clinical utility of the identified biomarkers and provide a foundation for future validation studies.

### LUSC cancer stage classification experiment

#### Data preprocessing

**Fig. 23:**
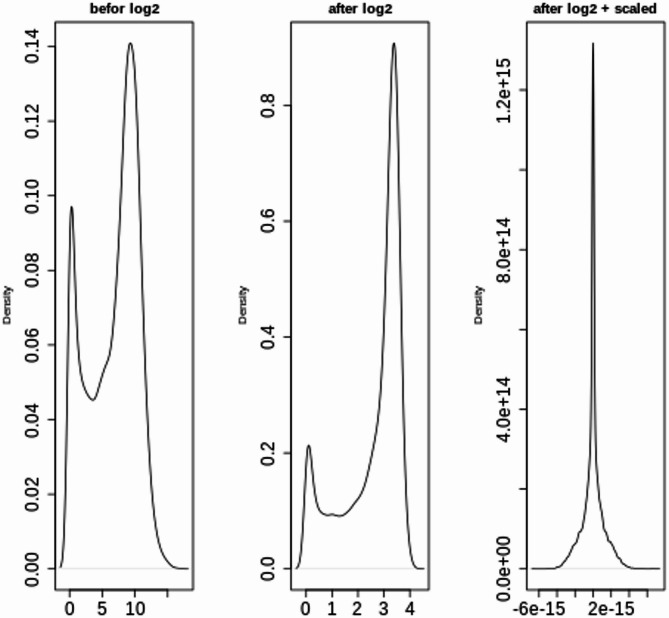
Distribution of RNA-seq data before and after logarithmic and scaling transformations

**Fig. 24:**
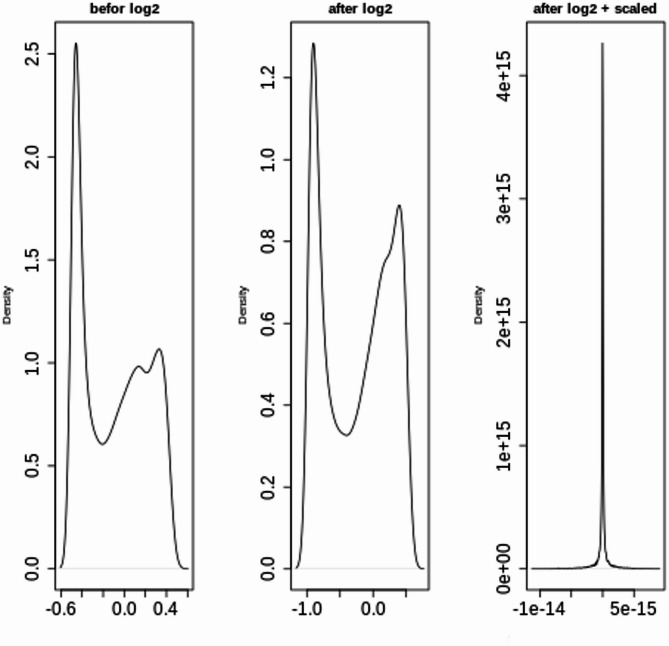
Distribution of methylation data before and after logarithmic and scaling transformations.

In this experiment, the proposed lung cancer stage classification model was applied to the TCGA-LUSC dataset to classify samples into Stage One and Stage Two. This experiment exclusively utilized RNA-seq data and methylation data. The preprocessing steps included data cleaning, filtration, and imputation of missing values, followed by normalization via logarithmic transformation and scaling for both types of tic data individually. The impact of the preprocessing steps on the data distribution are illustrated in two figures. Figures 23 and 24 shows the distributions of the RNA-seq and methylation data before and after transformation. In both panels, the first plot shows the original data distribution before any transformation, the second plot displays the distribution after applying a logarithmic (log2) transformation, and the third plot demonstrates the distribution after both log transformation and scaling. These transformations were applied to normalize the data, with the log transformation reducing skewness and scaling standardizing the distribution, thereby facilitating more accurate downstream analysis.

An analysis of each plot is provided below:

5. Before log2 (left plot):

The data distribution is shown before any transformations are applied. A multimodal distribution is observed, indicating the presence of multiple peaks within the dataset. A broad range of values is evident, likely due to skewness or the presence of large outliers.

6. After log2 (middle plot):

This plot displays the data following the application of a logarithmic transformation with base 2. The transformation reduces the impact of large values, resulting in a more condensed range. Although the peaks remain visible, the overall scale of the data is reduced, and the right skewness is diminished.

7. After log2 + scaling (right plot):

In this plot, the data have undergone both log transformation and scaling. Scaling further normalizes the data, adjusting its range and variance. A sharp peak is observed, suggesting that the data have been heavily condensed around a specific central point. The y-axis shows a much larger range, likely due to the scaling process.

The progression from left to right demonstrates the effects of applying transformations and scaling to a dataset to increase its suitability for further analysis, such as for machine learning models. The log transformation reduces skewness and compresses the range of values, while scaling further standardizes the data.

#### LUSC proposed models

Two complementary experiments were conducted on the TCGA-LUSC cohort. RNA-seq (406 samples; Stage I = 244, Stage II = 162) and DNA methylation (307 samples; Stage I = 172, Stage II = 135) were processed separately, and only patients with both omics were retained for integration (*n* = 307).

##### Model 1 — late-fusion CNN model

Feature selection was first performed independently within each omics type using XGBoost, yielding 293 RNA-seq features and 628 methylation features. The 307 common patients were then aligned, and the selected features were fused (921 features) to form the model input. A CNN classifier trained on this fused set achieved 82.26% accuracy, F1-score of 84.0%, and AUC 84% for Stage I vs. Stage II discrimination, indicating that multiomics late fusion provided substantial gains over single-omics models.

**Fig. 25:**
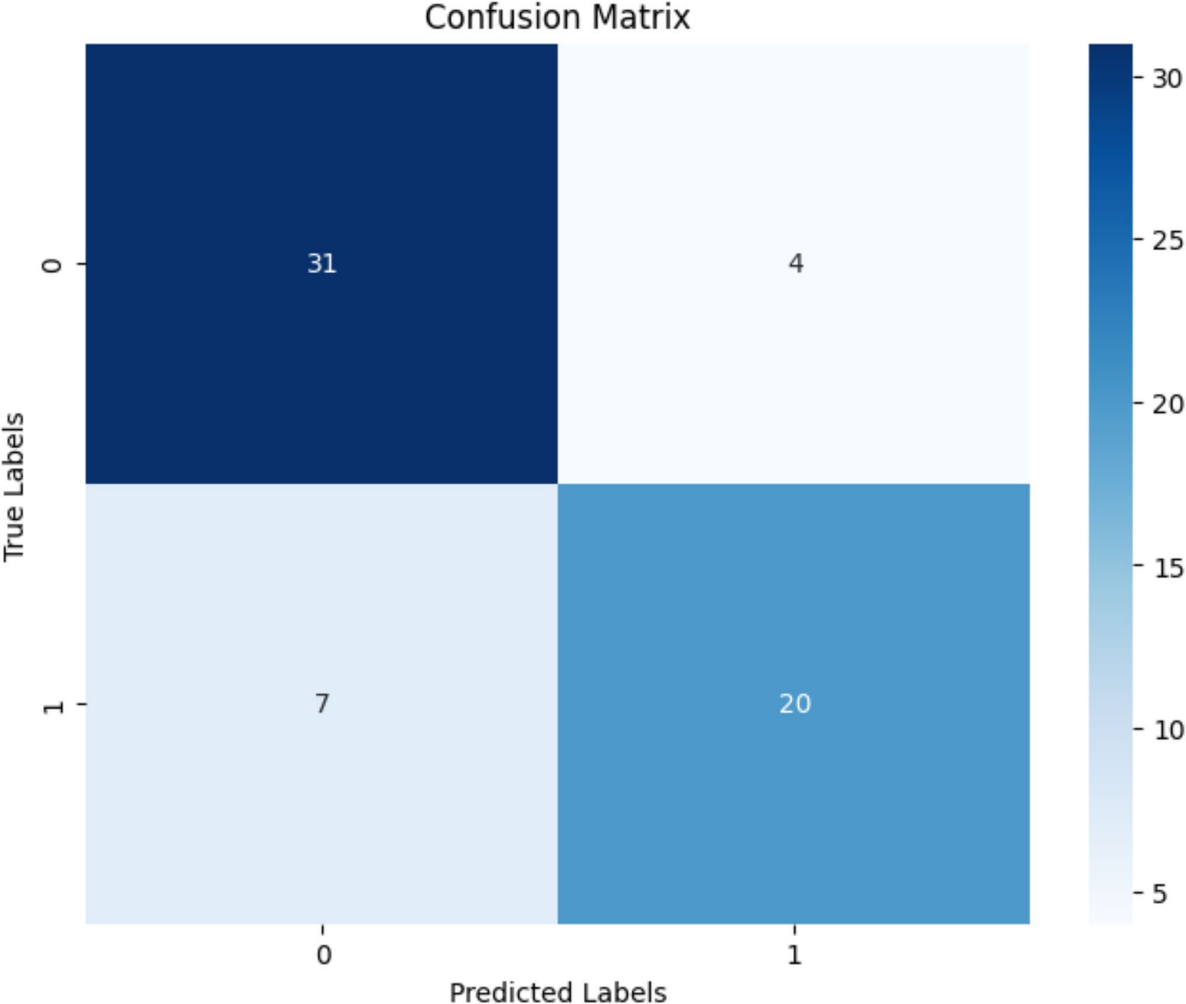
Confusion Matrix for the the XGB + DL LUSC stage classification model1 based on multiomics integration

The performance of the multiomics classification model, integrating RNA-seq and methylation data, was evaluated using a confusion matrix, as illustrated in Fig. 25. The matrix summarizes the number of correct and incorrect predictions made by the model, categorized by actual and predicted classes. A total 51 out of 62 instances were correctly classified, resulting in an overall accuracy of approximately82.26%. Specifically, 31 true negatives and 20 true positives were observed, while 4 false positives and 7 false negatives were recorded. This evaluation highlights the model’s capability to effectively distinguish between cancer subtypes based on integrated omics features.

**Fig. 26:**
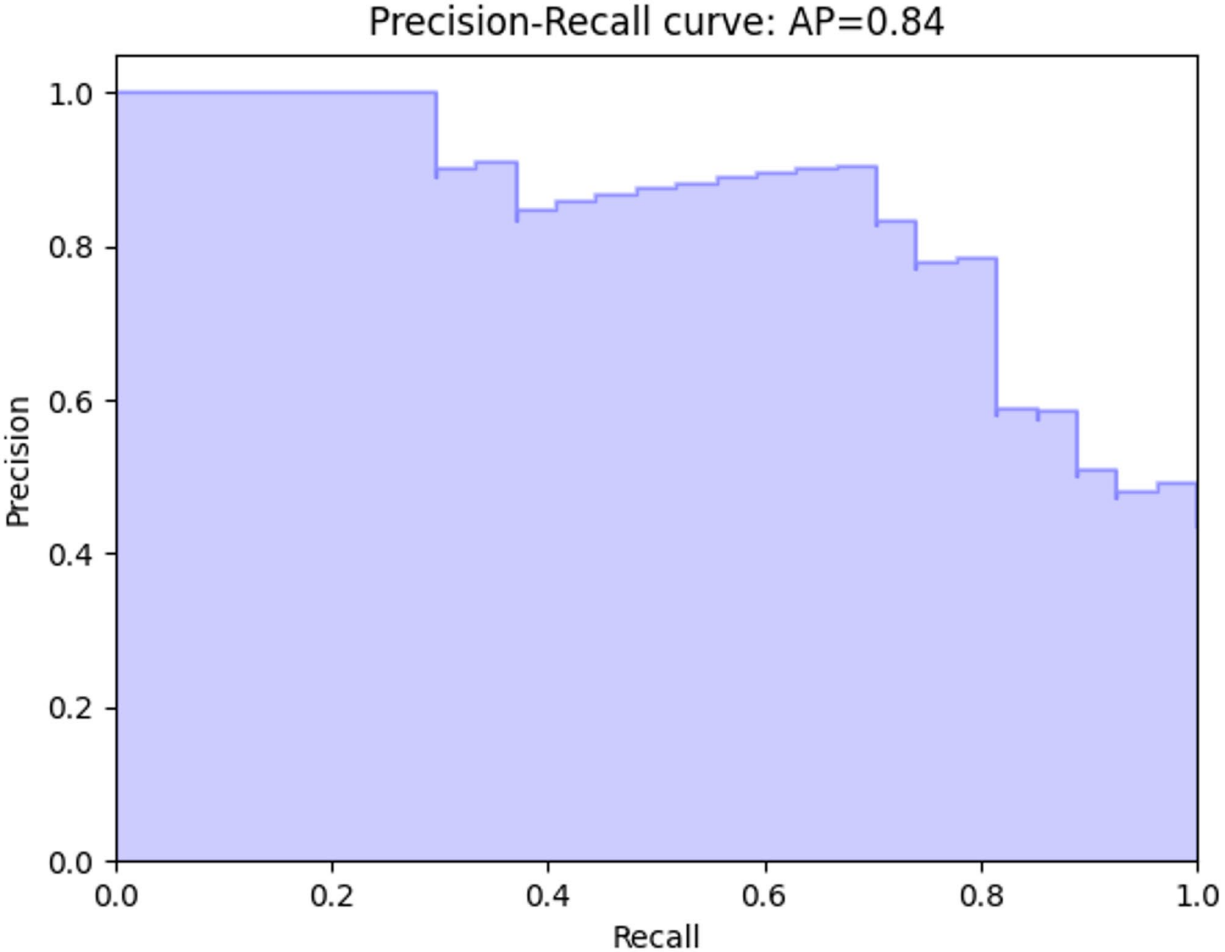
Precision-recall curve for the XGB + DL LUSC stage classification model1 based on multiomics integration

**Fig. 27:**
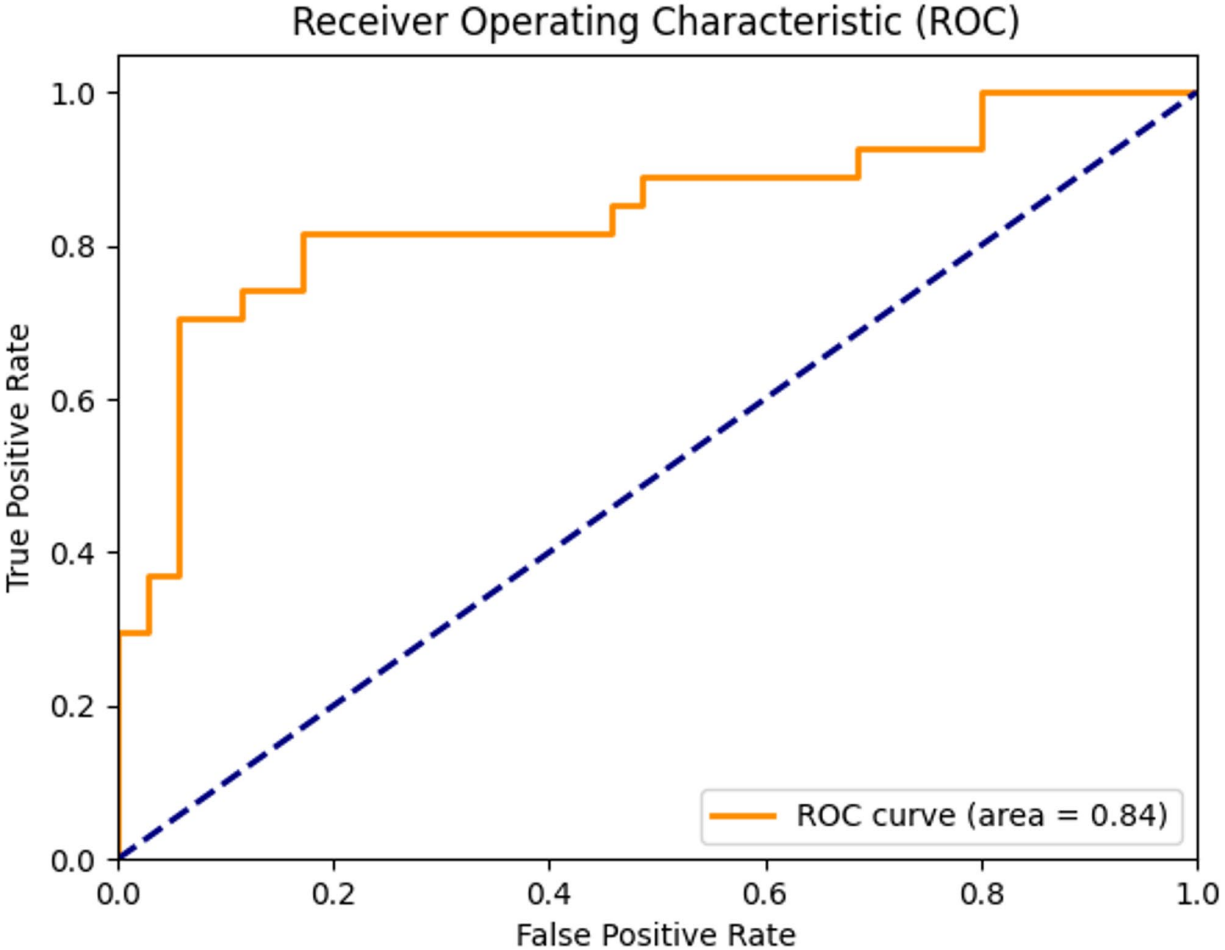
ROC curve for the XGB + DL LUSC stage classification model1 based on multiomics integration

To evaluate the LUSC stage classification model, precision–recall and ROC curves were generated. The precision–recall curve Fig. 26 showed high precision across recall levels with an average precision (AP) of 0.84, indicating effective handling of class imbalance. The ROC curve Fig. 27 yielded an AUC of 0.84, confirming strong discriminative power between Stage I and Stage II samples.

These complementary metrics demonstrate that the model balances precision and sensitivity, even under challenging class distributions. Importantly, the results indicate that integrating RNA-seq and methylation data enhances stage-specific signal detection compared to single-omics baselines. Additionally, it provides evidence for the robustness of the proposed framework in addressing clinically relevant classification tasks. These results highlight the robustness of integrating RNA-seq and methylation data with deep learning for reliable stage classification.

##### Model 2 — sDCFE hyper model

Feature selection in Model 2 was performed using sDCFE on the fused RNA-seq and methylation dataset (921 features after late fusion). The sDCFE cutoff retained 461 highly discriminative features, which were subsequently used as input to a logistic regression classifier. This dimensionality reduction strategy improved interpretability while maintaining robust classification performance. The model achieved an accuracy of 88.5% for Stage I vs. Stage II discrimination, 89% AUC, and 89% F1-Scoresurpassing the CNN-based Model 1 and highlighting the effectiveness of statistical feature selection in combination with a simpler classifier.

**Fig. 28:**
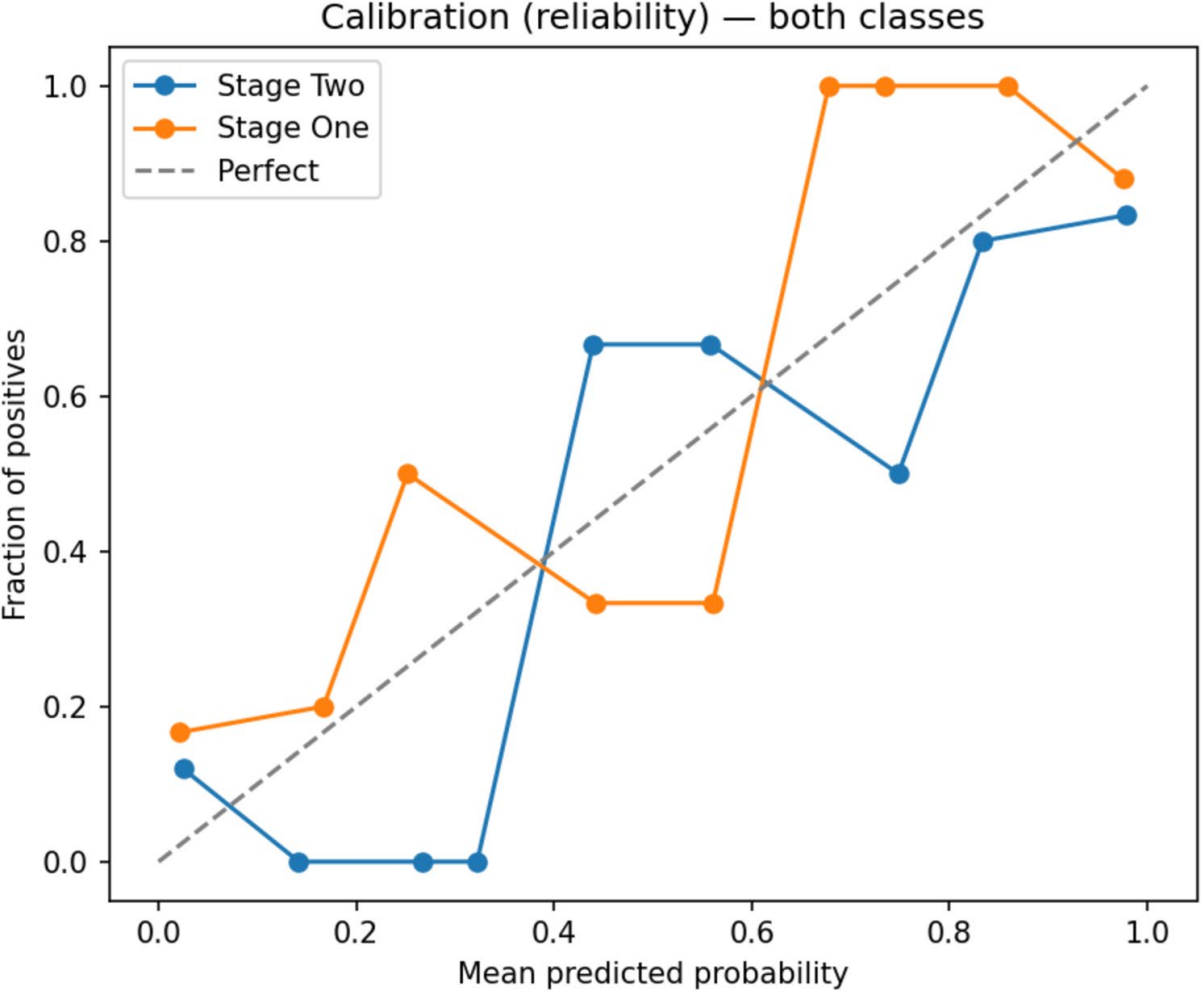
Calibration plot for the sDCFE + logistic regression LUSC stage classification model

To assess probability calibration, a calibration plot was generated Fig. 28. The curves for both Stage I and Stage II were closely aligned with the diagonal reference line, demonstrating that predicted probabilities corresponded well with observed outcomes. This indicates that the logistic regression model produced reliable probability estimates, an important property for clinical decision-making.

**Fig. 29:**
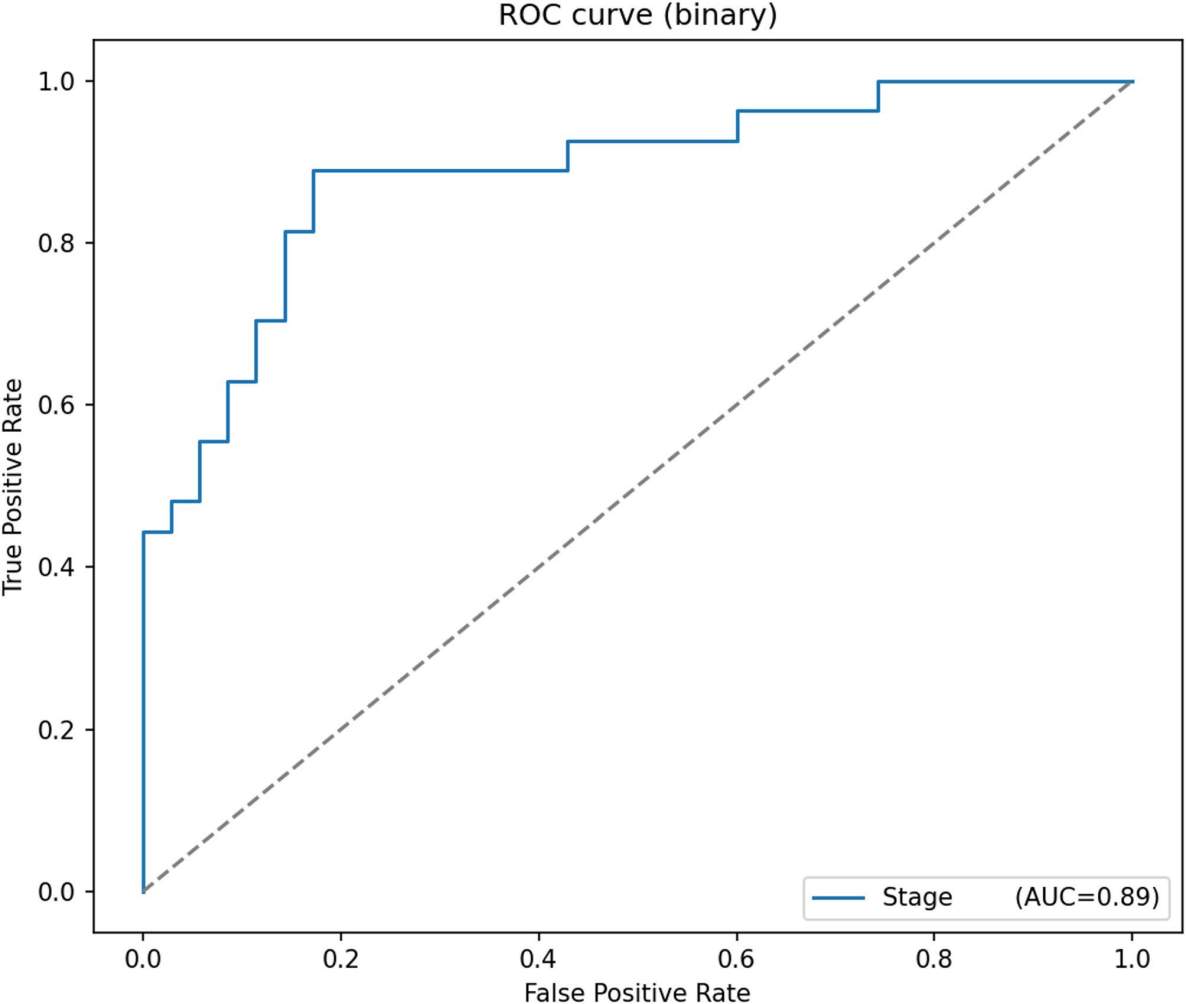
ROC curve for the sDCFE + logistic regression LUSC stage classification model (AUC = 0.89)

The discriminative ability of the model was further evaluated using ROC analysis Fig. 29. The ROC curve yielded an AUC of 0.89, reflecting strong separation between Stage I and Stage II patients. Compared to Model 1, the higher AUC and improved calibration underscore the advantage of combining sDCFE-based feature selection with logistic regression for multiomics stage classification. Together, these results establish Model 2 as a parsimonious yet accurate framework, with better calibrated predictions than the deep learning alternative.

**Fig. 30:**
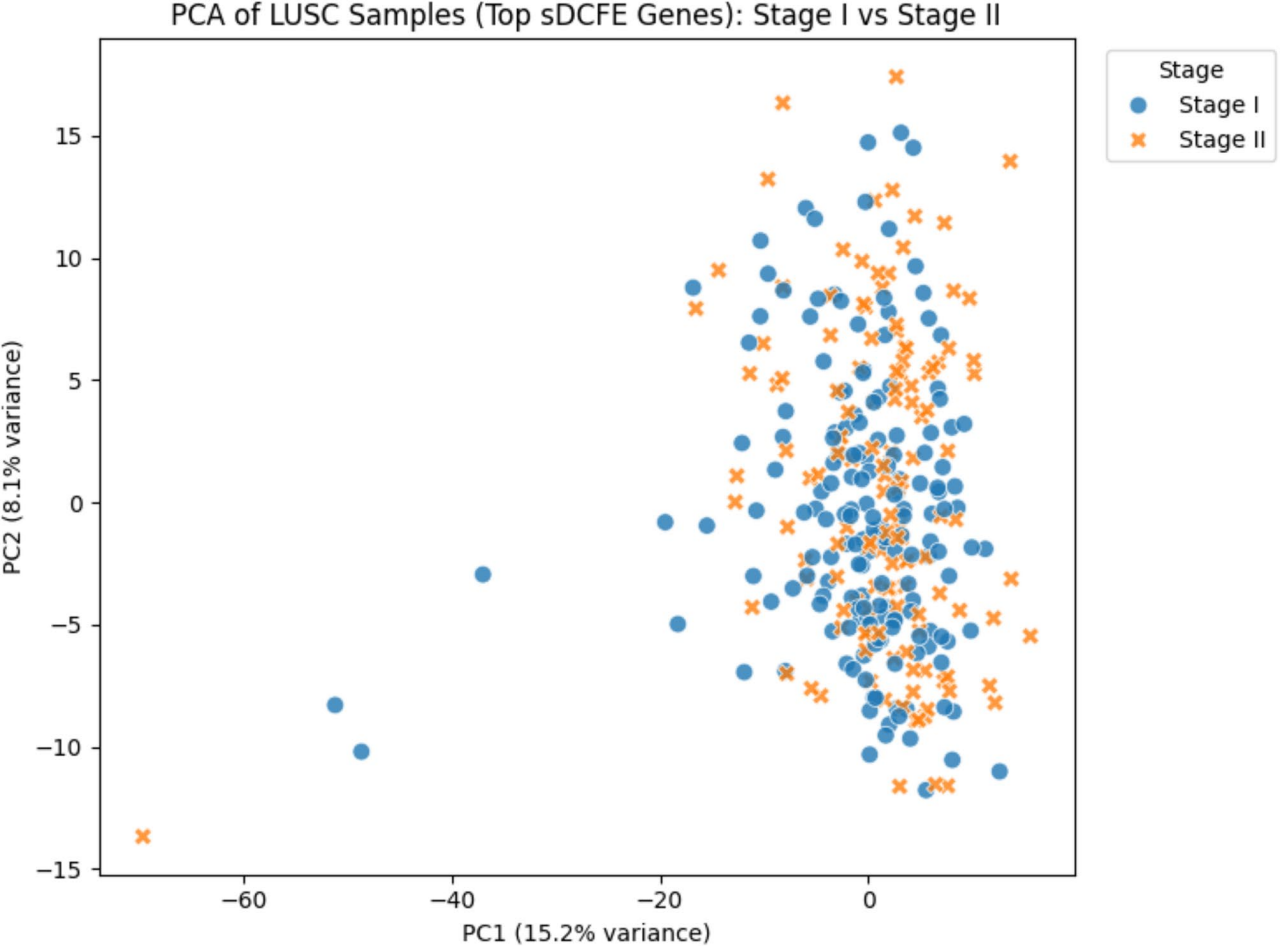
PCA of LUSC samples using the 461 sDCFE-selected genes

A PCA plot Fig. 30 generated using the 461 sDCFE-selected genes demonstrated partial separation between Stage I and Stage II. While overlap was evident, distinct variance captured along PC1 and PC2 indicated that sDCFE preserved meaningful stage-related structure. This visualization is consistent with the strong classification accuracy achieved by downstream models, confirming that the selected features retain biologically relevant signals associated with tumors progression. For context, single-omics XGBoost baselines yielded 60.16% accuracy on RNA-seq (293 features) and 76.0% on methylation (628 features), underscoring the advantage of both multiomics integration and sDCFE-guided refinement. Table 3 presents Summary of LUSC staging experiments. Collectively, these findings highlight the stability of sDCFE-selected features across different modelling strategies. Moreover, the balance of accuracy, calibration, and interpretability suggests that Model 2 may provide a practical solution for translational applications in cancer staging.

**Table 3:** Summary of feature selection and classification performance across RNA-seq, Methylation, and fused models

Data Type	Total Samples	Stage I	Stage II	Original Features	Features Selected (XGBoost)	Features Selected (sDCFE)	Classifier	Accuracy (%)
RNA-seq	406	244	162	20,104	293	-	XGBoost	60.16
Methylation	307	172	135	20,104	628	-	XGBoost	76
Model 1	307	172	135	921 (after late fusion)	-	-	CNN (DL)	84%
Model 2	307	172	135	921 (after late fusion)	-	461	Logistic Regression	88.50%

## Discussion

### Pancancer classification experiments

#### Generalizability of the hybrid model: TCGA performance and PCAWG validation

The hybrid framework, integrating XGBoost and sDCFE for feature selection with a deep learning classifier, demonstrated strong performance in pancancer classification across ten cancer types. On the TCGA test set, an accuracy of 99.3% was achieved, while external validation on the PCAWG cohort confirmed generalizability with an accuracy of 92%. These results indicate that the integration of sDCFE and XGBoost enabled the identification of highly discriminative features, which not only supported accurate classification but also provided insights into candidate biomarkers. The robustness of this framework suggests potential translational applications in stratified diagnostics and targeted therapies.

The discriminative variance component of sDCFE is closely related to Fisher score, which is widely used in supervised feature selection. The distinction lies in our inclusion of MAD-based regularization to penalize unstable features and the integration of cluster separation, which together provide greater stability and interpretability in noisy multiomics data. Thus, sDCFE may be viewed as a synergistic extension of Fisher-like methods rather than a replacement.

#### TCGA cohort: classification performance and visualization

Near-perfect discrimination was observed across the ten TCGA cancer types. The ROC curves Fig. 6 achieved AUC values of 1.0 for all classes, with each curve tightly aligned with the top-left corner, reflecting minimal false positive rates and near-maximal sensitivity. Calibration analysis Fig. 7 further confirmed reliability, as probability estimates for most cancer types closely followed the ideal calibration line. Minor deviations, such as slight overconfidence for STAD and LIHC, were observed but did not substantially affect reliability.

The row-normalized confusion matrix (Fig. 8) further summarized the per-class prediction results. Overall, the majority of cancer types were classified with near-perfect accuracy, with values close to 1.00 along the diagonal. Only limited misclassification was observed, such as a small proportion of UCEC samples predicted as STAD (0.03) and THCA samples predicted as LUAD (0.01). Importantly, because each row was normalized to 100%, the shading in the matrix reflected the relative classification accuracy per cancer type rather than being influenced by differences in dataset size. This representation confirms the robustness of the model across all ten cancer types while ensuring fair comparability between larger cohorts (e.g., COADREAD, 629 samples) and smaller cohorts (e.g., KIRP, 291 samples).

In addition, PCA visualization Fig. 9 using the 350 sDCFE-selected genes confirmed that biological separability was preserved. Distinct clusters were observed for LIHC, LGG, and THCA, while UCEC and PRAD also formed compact groups. Partial overlap was seen for BLCA and COADREAD, consistent with their molecular heterogeneity. This unsupervised clustering analysis corroborated the supervised classification metrics, highlighting the discriminative power of sDCFE features in capturing biologically meaningful variance.

#### PCAWG cohort: external validation

The PCAWG cohort was used to evaluate the external generalizability of the framework. The one-vs-rest ROC curves (Fig. 10) yielded AUC values ranging from 0.99 to 1.00. Perfect discrimination (AUC = 1.00) was observed for THCA, PRAD, LIHC, KIRP, and LGG, while UCEC (AUC = 0.990), STAD (AUC = 0.997), COADREAD (AUC = 0.998), LUAD (AUC = 0.999), and BLCA (AUC = 0.994) showed slightly lower, but still very strong, performance. The calibration performance Fig. 11 appeared more variable compared to TCGA. While several cancer types, such as LUAD, THCA, and LGG, remained relatively close to the ideal calibration line, others including STAD, LIHC, and KIRP exhibited pronounced fluctuations. These deviations are likely attributable to the reduced number of informative features available in the external PCAWG cohort, as well as smaller per-class sample sizes, both of which can affect the stability of probability calibration.

The row-normalized confusion matrix Fig. 12 indicated strong per-class predictions across PCAWG, with correct classifications dominating the diagonal. The row-normalized confusion matrix (Fig. 12) shows that correct classifications dominate the diagonal across PCAWG. PRAD, LIHC, and LGG achieved perfect accuracy (1.00), while KIRP and COADREAD were near-perfect (0.98). THCA reached 0.94 and UCEC 0.90, whereas BLCA and LUAD obtained 0.83 and 0.80, respectively; STAD was lower at 0.77. The observed reduction in classification performance is primarily attributable to the absence of a subset of features in the independent cohort. While the framework was originally trained with 1,205 selected features, only 905 of these were available in the PCAWG dataset. The missing features included several discriminative genes that the model had previously relied upon, thereby reducing classification accuracy for certain cancer types. Nevertheless, the framework maintained strong predictive power across most cohorts, demonstrating robustness even under conditions of incomplete feature availability. Misclassifications were limited and mainly involved such clinically or biologically related cancers. Although overall performance was marginally lower than in TCGA, the framework maintained strong predictive power, and the use of row-normalized matrices ensured fair comparison across cancer types regardless of sample size.

#### Baseline comparisons and feature discriminability

Baseline comparisons confirmed the strength of the hybrid framework Table 1. XGBoost alone achieved high predictive accuracy, while sDCFE alone yielded slightly lower values, consistent with its role as a feature selection method rather than a classifier. Logistic Regression and SVM, when applied to sDCFE-selected features, achieved accuracies comparable to XGBoost, confirming the discriminative value of the 350 sDCFE genes.

**Fig. 31:**
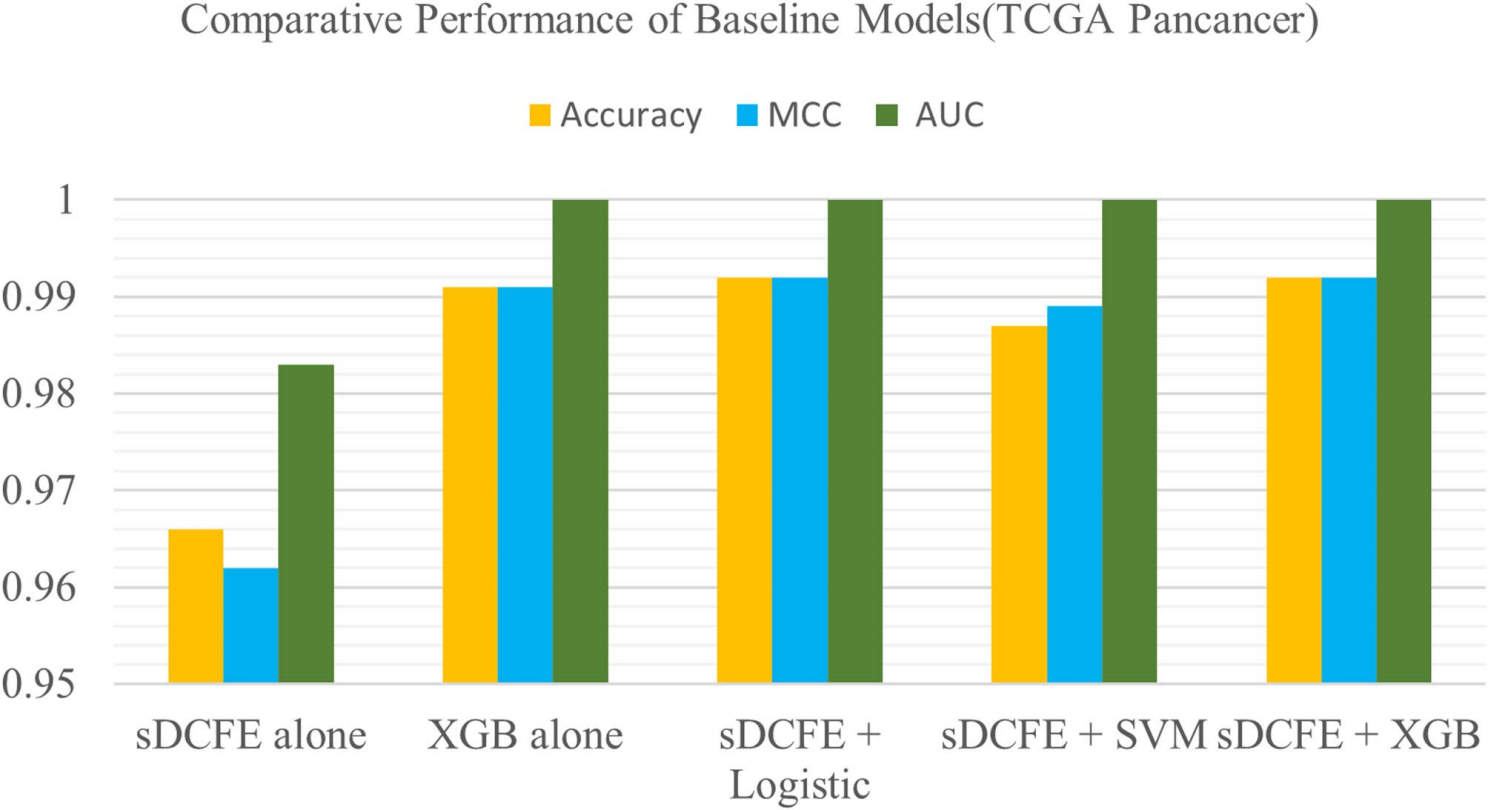
Performance benchmark of sDCFE with logistic regression, SVM, and XGBoost (TCGA Pancancer)

The combined sDCFE + XGBoost model achieved performance levels similar to XGBoost alone, with the added benefit of enhancing feature stability and interpretability. The close performance of simpler models such as Logistic Regression indicated that classification success was driven primarily by the informative features rather than classifier complexity. A comparative bar chart Fig. 31 summarizes these results, underscoring that the integration of sDCFE enhances robustness without compromising performance.

#### Functional enrichment of intersected genes

Functional enrichment analysis of the 82 intersected genes revealed their involvement in key biological pathways Fig.16. GO BP terms included thyroid gland development, hormone secretion regulation, and acute-phase response, linking the gene set to endocrine signalling and systemic inflammatory activity. Additional enriched processes such as heme catabolism and plasminogen activation reflected roles in metabolic adaptation and extracellular matrix remodelling.

Reactome analysis Fig. 15 further highlighted pathways including amino acid conjugation, complement activation, and lipid remodelling, emphasizing contributions to metabolic and immune regulation. KEGG analysis Fig. 14 identified enrichment in complement and coagulation cascades, thyroid hormone synthesis, and calcium signalling pathways, all of which are directly implicated in angiogenesis, immune evasion, and hormone-driven oncogenesis. Collectively, these findings confirmed that the intersected gene set represents biologically meaningful markers rather than random statistical associations.

#### Biomarker novelty and translational relevance

The novelty assessment was performed to stratify the 82 intersected genes into three categories: established biomarkers, emerging candidates, and novel genes. A total of 12 genes were confirmed as established biomarkers, with support obtained from multiple curated resources including COSMIC, OncoKB, and HPA. A larger subset of 64 genes was classified as emerging, as annotations were found only in broad resources such as HPA or DisGeNET but were absent from more stringent biomarker databases. In addition, six genes (HFE2, LOC339674, SERINC2, SFTA3, SOX2OT, and ACPP) were identified as novel, with no prior biomarker annotations reported.

**Fig. 32:**
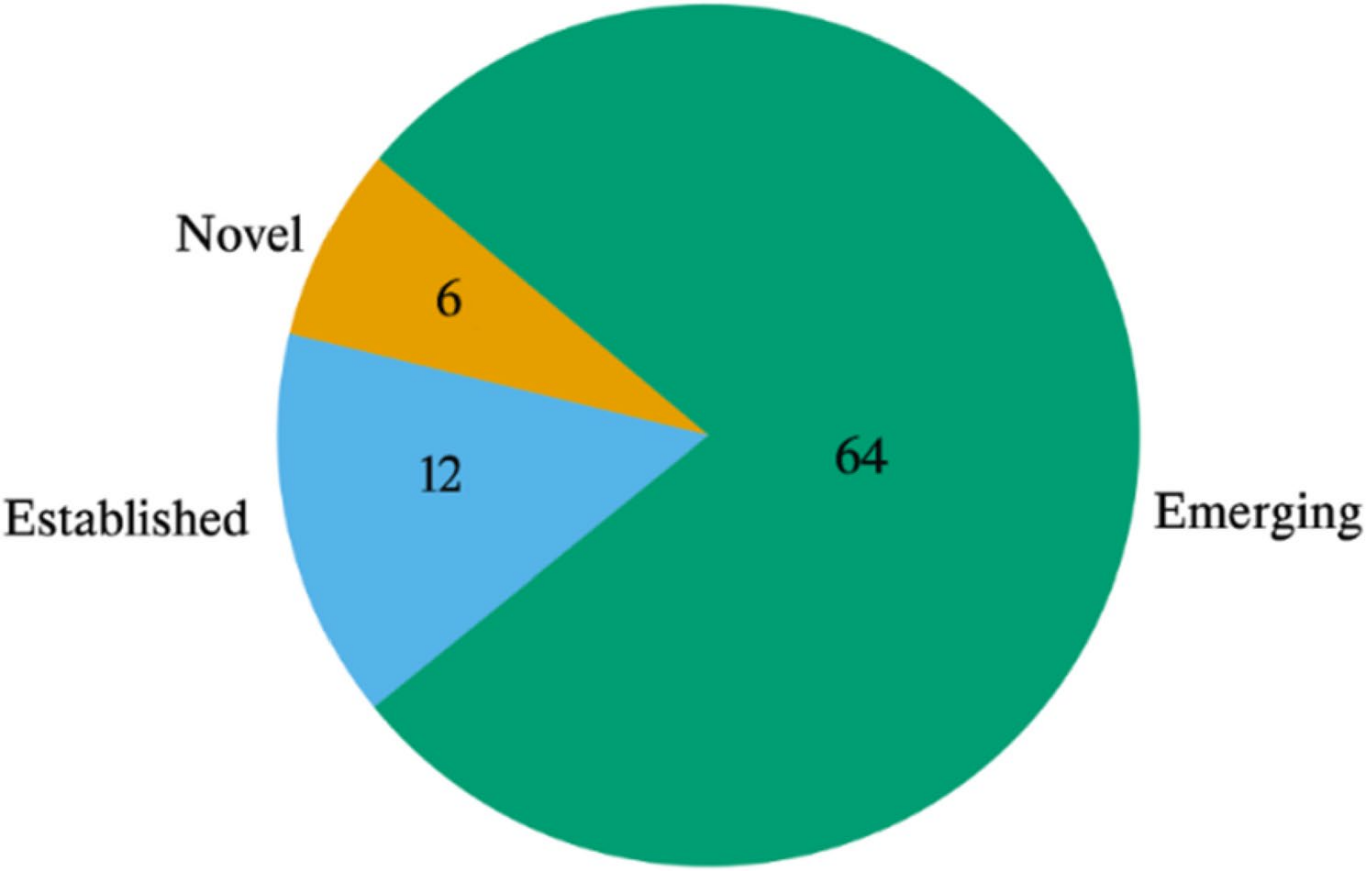
Distribution of biomarkers by evidence category

Through this classification, the ability of the proposed framework to rediscover established biomarkers, highlight underexplored candidates, and prioritize novel genes for validation was demonstrated, thereby broadening the landscape of potential translational targets. The distribution of these categories is illustrated in Fig. 32 (pie chart), and the complete classification, including per-gene evidence across six databases and calculated mean scores, is reported in Supplementary Table S2.

#### Prognostic Relevance of Identified Biomarkers

Survival analysis strengthened the clinical relevance of the identified biomarkers. In LUAD, SFTA3 exhibited a strong protective effect, while ACPP also showed protective associations. In LIHC, HFE2 correlated with improved survival, whereas LOC339674 was linked to adverse prognosis. In LGG, high SERINC2 expression was significantly associated with worse outcomes, while LOC339674 demonstrated a very strong protective effect. In KIRP, LOC339674 again showed adverse associations, while in COADREAD, SOX2OT expression was linked to poorer survival.

Six representative KM plots Figs. 17, 18, 19, 20, 21 and 22 illustrated these associations, confirming that the biomarkers captured prognostic signals across distinct cancer types. The ability to link classification-derived features with survival outcomes enhances translational impact by moving beyond diagnostic utility to clinical outcome prediction.

#### Comparison with existing studies

When compared with prior studies, including Almuayqil et al. [8], the proposed framework demonstrated superior performance. While both studies utilized advanced machine learning approaches, the present model achieved higher accuracy (99.6% vs. lower reported values) across a larger set of cancer types. The combination of statistical and model-driven feature selection, coupled with deep learning classifiers, enabled superior generalizability and interpretability. These findings establish the proposed hybrid framework as a state-of-the-art method for pancancer classification and biomarker discovery.

### LUSC Cancer stage classification experiment

#### Model 1— late-fusion CNN model

LUSC stage classification was first performed by integrating RNA-seq and methylation data through a late-fusion deep learning framework. Feature selection was independently carried out for each omics type using the XGBoost algorithm, which retained 293 RNA-seq features and 628 methylation features. A total of 307 patients with both RNA-seq and methylation profiles were then aligned, and the selected features were merged, yielding 921 integrated features. This fused dataset was subsequently used to train a convolutional neural network (CNN) classifier for Stage I versus Stage II discrimination.

The performance of the CNN-based late-fusion model for LUSC stage classification was comprehensively evaluated using multiple complementary metrics and visualizations. The confusion matrix Fig. 25 demonstrated that the model achieved reliable stage discrimination, with 31 Stage I samples and 20 Stage II samples correctly classified. Only a limited number of misclassifications were observed (4 false positives and 7 false negatives), confirming the model’s overall ability to capture discriminative patterns between stages.

The precision–recall (PR) curve Fig. 26 further illustrated this balance, with an average precision (AP) score of 0.84. This indicates that the model was able to maintain high precision across a range of recall levels, an especially important property given the moderate class imbalance in the dataset. High PR performance therefore reinforces that the model minimizes false positives while still retrieving a large proportion of true Stage II cases.

The ROC curve Fig. 27 provided complementary evidence of robustness, yielding an area under the curve (AUC) of 0.84. The ROC highlights the strong trade-off achieved between sensitivity and specificity, confirming the discriminative power of the model in separating early- and mid-stage tumors.

These results indicate that the CNN-based late-fusion framework effectively integrated RNA-seq and methylation features to capture stage-related biology. The confusion matrix highlights reliability at the sample level, the PR curve demonstrates robustness under imbalanced conditions, and the ROC curve confirms overall discriminative ability. The convergence of these findings validates the model as a reliable tool for stage prediction in LUSC, showing that deep learning applied to multiomics integration can extract clinically meaningful signals.

#### Model 2 — sDCFE hyper model

In the second experiment, the integrated feature set of 921 variables was further refined using the sDCFE framework. Features were ranked according to their discriminative scores, and the top 461 were selected. Logistic regression with L2 regularization was then applied to the reduced dataset.

The evaluation of Model 2 demonstrated that the integration of sDCFE-based feature selection with logistic regression yielded not only high predictive performance but also strong reliability. The ROC curve Fig. 29 illustrates a robust discriminative capacity, achieving an AUC of 0.89, which indicates that the model effectively distinguishes between Stage I and Stage II LUSC cases with minimal overlap between true positives and false positives. Complementing this, the calibration plot Fig. 28 showed that the predicted probabilities were closely aligned with observed outcomes for both stages, reflecting clinically meaningful reliability. The proximity of the stage-specific curves to the diagonal “perfect calibration” line suggests that the model produces well-calibrated risk estimates, an essential property for translational applications where accurate probability outputs is critical for patient stratification.

The PCA visualization Fig. 30 provided an unsupervised validation of the sDCFE-selected feature space. Although Stage I and Stage II samples exhibited partial overlap, a clear trend of separation along PC1 was observed, indicating that the selected features preserved biologically meaningful stage-related variance. Importantly, the clustering tendencies evident in PCA align with the strong discriminative performance shown in the ROC analysis, while the calibration plot further confirms that these stage-related patterns were translated into reliable probability estimates by the logistic regression classifier. Taken together, these complementary findings underscore that Model 2 not only identified informative features with sDCFE but also translated them into accurate, well-calibrated predictions, offering both statistical robustness and biological interpretability for LUSC stage classification.

#### Comparison with lternative integration strategies

In this study, feature selection was performed separately for RNA-seq and methylation datasets, followed by late fusion to construct the integrated models. This approach ensured that the most relevant signals from each omics layer were retained before classification. By contrast, Zhao et al. [53] proposed an early-fusion strategy in which different omics measurements for the same gene were concatenated into a multi-dimensional feature vector. Such an approach enables the joint evaluation of cross-omics interactions but substantially increases dimensionality and requires larger sample sizes and stronger regularization to mitigate overfitting.

In contrast to the work of Alina [25], who developed a hybrid deep neural network using open-source libraries such as TensorFlow and Keras, the methodology proposed in this study was designed to incorporate a more comprehensive feature selection phase. Alina’s models—DBN-ELM-BP and DBN-ELM-ELM—were constructed via a combination of genetic algorithms, extreme learning machines (ELMs), and deep belief networks (DBNs) and were evaluated on TCGA datasets comprising mRNA expression, miRNA, DNA methylation, and clinical features. In the current study, a broader set of discriminative features was leveraged, which may have contributed to the improved classification accuracy. A comparative summary of classification performance across existing models is presented in Table 4, where the proposed model was shown to outperform previous approaches in terms of both accuracy and robustness.

As summarized in Table 4, the proposed models outperformed several prior methods, demonstrating superior accuracy and calibration. These findings indicate that the hybrid XGBoost + sDCFE framework provides a robust and generalizable solution for LUSC stage classification, while also maintaining interpretability and stability of the selected features.

**Table 4:** Comparative performance analysis of LUSC cancer stage classification methylation and RNA-seq data

Methods	Accuracy (%)	F1-score (%)
WE-DBN [25]	68	72.93
DBN-ELM [25]	77.87	75.69
DBN-ELM-BP (Methylation) [25]	83.61	63.56
DBN-ELM-BP (mRNA) [25]	75.23	65.34
DNN [25]	78.08	77.08
Google net [25]	75.66	75.21
Proposed model 1	82.26	84.0
Proposed model 2	88.5%	89.0

## Conclusions

In this study, a novel feature selection method, sDCFE, was developed and demonstrated to be a statistically principled and biologically informative approach for high-dimensional omics data. When integrated with XGBoost and deep learning, it enabled state-of-the-art pancancer classification, achieving 99.3% accuracy on TCGA and 94% accuracy on PCAWG, thus confirming strong generalizability. In parallel, the LUSC stage classification models highlighted the importance of multiomics integration: a CNN-based model reached 84% accuracy, while an sDCFE + Logistic Regression model improved interpretability and calibration, achieving 88.5%. These results underscore the robustness of the hybrid framework across both broad pancancer and focused stage-specific applications, outperforming several state-of-the-art approaches.

Beyond classification performance, sDCFE facilitated the identification of meaningful biomarkers. Enrichment analysis confirmed that the 82 intersected genes were strongly involved in cancer-relevant pathways, while survival analysis highlighted six robust biomarker–cancer associations: LUAD–SFTA3 (protective), LUAD–ACPP (protective), LIHC–HFE2 (protective), LIHC–LOC339674 (adverse), LGG–SERINC2 (risk), and LGG–LOC339674 (strong protective). The discovery of these genes as potential prognostic biomarkers demonstrates the translational value of the pipeline in precision oncology.

Future work will therefore focus on expanding validation to additional independent cohorts, refining feature selection criteria with automated tuning strategies, and developing an extended sDCFE capable of directly operating on integrated multi-dimensional omics data. Moreover, prospective clinical validation and wet-lab experiments will be essential to confirm the functional relevance of the identified biomarkers and support their translation into real-world cancer diagnostics and treatment.

## Supplementary Information

Supplementary Material 1

Supplementary Material 2

Supplementary Material 3

## Data Availability

The datasets used and analysed during the current study are available in publicly accessible repositories. Specifically, data were sourced from The Cancer Genome Atlas (TCGA) [https://portal.gdc.cancer.gov/](https:/portal.gdc.cancer.gov/%20) and Pancancer Analysis of Whole Genomes (PCAWG) project [https://www.nature.com/articles/s41586-020-1969-6](https:/www.nature.com/articles/s41586-020-1969-6), which is part of the ICGC/TCGA consortium Detailed information about the datasets utilized in this research can be found in the method section. All data are available from these repositories, and additional details can be requested from the corresponding author upon reasonable request.

## Abbreviations

sDCFE: Synergistic Discriminative Cluster-based Feature Extraction
MAD: Median Absolute Deviation
KNN: K-Nearest Neighbors
SVM: Support Vector Machine
XGBoost: Extreme Gradient Boosting
KM: Kaplan–Meier
KEGG: Kyoto Encyclopedia of Genes and Genomes
GO BP: Gene Ontology Biological Process
COSMIC: Catalogue of Somatic Mutations in Cancer
OncoKB: Precision Oncology Knowledge Base
CIViC: Clinical Interpretations of Variants in Cancer
ONGene: Oncogene Database
HPA: Human Protein Atlas
DisGeNET: Disease–Gene Associations Database
ACC: Accuracy
MCC: Matthews Correlation Coefficient
OvR: One-vs-Rest
ECE: Expected Calibration Error
PR: Precision–Recall
F1: F1-Score, harmonic mean of precision and recall
NS: Not Significant
sig: Significant
DL: Deep Learning
ML: Machine Learning
CNN: Convolutional Neural Network

## References

[1] World Health Organization. Global cancer statistics, 2020.

[2] Siegel RL, Miller KD, Jemal A. Cancer statistics, CA: A cancer. J Clin. 2020;70(1):7–30. 10.3322/caac.21590 31912902

[3] National Cancer Institute. Cancer screening overview, 2019.

[4] Reham Ashraf Shafik; Mahmoud Monir; Yasmine M. Afify; Nagwa Badr."Genetic Biomarkers Detection for Alzheimer’s Disease**", International Jo urnal of Intelligent Computing and Information Sciences. 2025;25(1):51-73. 10.21608/ijicis.2025.375039.1388.

[5] Wang Z, Gerstein M, Snyder M. RNA-Seq: a revolutionary tool for transcriptomics. Nat Rev Genet. 2009;10(1):57–63. 19015660 10.1038/nrg2484 PMC2949280

[6] Rahnenführer, J., De Bin, R., Benner, A., “Statistica analysis of high-dimensional biomedical data: a gentle introduction toanalytical goals, common approaches and challenges, “BMC Med 21, 182, 2023. 10.1186/s12916-023-02858-y. 10.1186/s12916-023-02858-y PMC10186672 37189125

[7] Ghaleb MS, Maryam N. Al-Berry, Hala M. Ebied, and Mohamed F. Tolba. “Ovarian CancerProteome Analysis and Biomarker Discovery Using Machine Learning, “10thInternational Conference on Advanced Intelligent Systems and Informatics(AISI’24), 2024. 10.1007/978-3-031-77299-3_2.

[8] Almuayqil,S.N.; Elbashir, M.K.; Ezz, M.; Mohammed, M.; Mostafa, A.M.; Alruily, M.; Hamouda, E. An Approach for Cancer-Type Classification Using Feature SelectionTechniques with Convolutional Neural Network. Appl. Sci. 2023;13:10919. 10.3390/app131910919.

[9] Ghaleb, M.S., Ebied, H.M., Tolba, M.F. (2023). Bladder Cancer Microarray Analysis and Biomarker Discovery Using Machine Learning. In: Hassanien, A., Rizk, R.Y., Pamucar, D., Darwish, A., Chang, KC. (eds) Proceedings of the 9th InternationalConference on Advanced Intelligent Systems and Informatics 2023. AISI 2023. Lecture Notes on Data Engineering and Communications Technologies, vol 184. Springer, Cham. 10.1007/978-3-031-43247-7_25

[10] Kursa MB, Rudnicki WR. Feature selection with the Boruta package. J Stat Softw Vol. 2010;36. 10.18637/jss.v036.i11.

[11] Saeys Y, Inza I, Larrañaga P. A review of feature selection techniques in bioinformatics. Bioinformatics. 2007. 10.1093/bioinformatics/btm344. 17720704 10.1093/bioinformatics/btm344

[12] Takefuji, Yoshiyasu, “Reassessingfeature importance biases in machine learning models for infection analysis,“Journal of Infection, vol: 89, Elsevier. 2025. 10.1016/j.jinf.2024.106357. 10.1016/j.jinf.2024.106357 39557091

[13] Lecun Y, Bengio Y, Hinton G. Deep learning. Nature. 2015;521:436-444. https://doi.org/10.1038/nature14539. 10.1038/nature14539 26017442

[14] Topol EJ. High-performance medicine: the convergence of human and artificial intelligence. Nat Med. 2015;25(1):44–56. 10.1038/s41591-018-0300-7 30617339

[15] Esteva A, Robicquet A, Ramsundar B, Kuleshov V, DePristo M, Chou K, et al. A guide to DL in healthcare. Nat Med. 2019;25(1):24–9. 30617335 10.1038/s41591-018-0316-z

[16] Chen Z-H, Lin L, Wu C-F, Li C-F, Xu R-H, Sun Y. Artificial intelligence for assisting cancer diagnosis and treatment in the era of precision medicine. Cancer Commun. 2021;41(11):1100–15. 10.1002/cac2.12215. 10.1002/cac2.12215 PMC8626610 34613667

[17] Hanan A, Howida S, Safwat H, Ashraf S, Convolutional neural network models, for cancer treatment response prediction., International Journal of Intelligent Computing and Information Sciences. 2023;23. 10.21608/ijicis.2023.180508.1239.

[18] Patil SB, Patil RV, Mahalle PN. Early breast cancer prediction using machine learning and deep learning techniques. Int J Recent Innov Trends Comput Communication. 2023;11(10 s):111–7. 10.17762/ijritcc.v11i10s.7603.

[19] Ghaleb MS, Maryam N, Al-Berry HM, Ebied, Tolba MF. Lung Cancer Stages Classification Based on Differential Gene Expression and DL, 10th International Conference on Advanced Intelligent Systems and Informatics (AISI’24). 2024.

[20] Chaudhary K, Poirion OB, Lu L, Garmire LX. Dl–based multiomics integration robustly predicts survival in liver cancer. Clin Cancer Re’search. 2018;24(6):1248–59. 10.1158/1078-0432.CCR-17-0853 PMC6050171 28982688

[21] Zeng Z, Mao C, Vo A, Li X, Nugent JO, Khan SA, Clare SE, Luo Y. DL for cancer type classification and driver gene identification. BMC Bioinformatics. 2021;22(Suppl 4):491. 10.1186/s12859-021-04400-4. PMID: 34689757; PMCID: PMC8543824. 34689757 10.1186/s12859-021-04400-4 PMC8543824

[22] Chakraborty S, Hosen MI, Ahmed M, Shekhar HU. “Onco-multiomics Approach, “A New Frontier in Cancer Research.Biomed Res Int. 2018. 10.1155/2018/9836256. PMID: 30402498; PMCID:PMC6192166. 10.1155/2018/9836256 PMC6192166 30402498

[23] Zhao J, Cheng W, He X, Liu Y, Li J, Sun J, et al. Construction of a specific SVM classifier and identification of molecular markers for lung adenocarcinoma based on lncRNA–miRNA-mRNA network. Onco Targets Ther. 2018;11:3129. 10.2147/OTT.S151121. 29872324 10.2147/OTT.S151121 PMC5975616

[24] Fan Z, Xue W, Li L, Zhang C, Lu J, Zhai Y, Identification of an early diagnostic biomarker of lung adenocarcinoma based on coexpression similarity and construction of a diagnostic model, J Transl Med, 16(1):205. 10.1186/s12967-018-1577-5 PMC6053739 30029648

[25] Alina A, Aysun C. Enhancing cancer stage prediction through hybrid deep neural networks: a comparative study, Machine Learning and Artificial Intelligence. 2024;7. 10.3389/fdata.2024.1359703 PMC10995364 38586474

[26] Ahmed Hesham El-Tohamy; Huda Amin;Nagwa Badr. "Integration of Deep Learning Models for EnhancedClassification of Viral DNA Sequences Across Specific Viruses and ViralFamilies*", **International Journal of Intelligent Computingand Information Sciences. *2024;24(1):89-104. 10.21608/ijicis.2024.279692.1332.

[27] Mucherino A, Papajorgji PJ, Pardalos PM. k-Nearest neighbour classification. Data mining in Agriculture, springer optimization and its applications. Volume 34. New York, NY: Springer; 2009. 10.1007/978-0-387-88615-2_4.

[28] Feng C, Wang H, Lu N, Chen T, He H, Lu Y, Tu XM. Log-transformation and its implications for data analysis. Shanghai Arch Psychiatry. 2014;26(2):105–9. 10.3969/j.issn.1002-0829.2014.02.009. 25092958 10.3969/j.issn.1002-0829.2014.02.009 PMC4120293

[29] Wickham H, Pedersen T, Seidel D. scales: Scale Functions for Visualization. R package version 1.3.0, 2023. https://github.com/r-lib/scales, https://scales.r-lib.org

[30] Tianqi C, Carlos G. XGBoost: a scalable tree boosting system. In proceedings of the 22nd ACM SINGKKD International conference on knowledge discovery and data mining (KKD '16) . Asssociation for computing machinery, New York, NY, USA. 2016:785–794. https:doi.org/10.1145/2939672.2939785.

[31] Cortes C, Vapnik V. Support-vector networks. Mach Learn. 1995;20(3):273–97.

[32] Cox DR. The regression analysis of binary sequences. J Roy Stat Soc: Ser B (Methodol). 1958;20(2):215–32.

[33] Chen EY, Tan CM, Kou Y, DuanQ, Wang Z, Meirelles GV, Clark NR, Ma’ayan A.,” Enrichr:interactive and collaborative HTML5 gene list enrichment analysis tool, “BMCBioinformatics. 2013;14:128. 10.1186/1471-2105-14-128. 10.1186/1471-2105-14-128 PMC3637064 23586463

[34] Fang et al., “GSEApy: a comprehensive Python package for geneset enrichment analysis, “ 2023.

[35] Yu,“Functional enrichment analysis was conducted using the clusterProfilerR package leveraging GO and KEGG ontologies to interpret gene clusters, “2012.

[36] Anehisa M, Furumichi M, Sato Y, Ishiguro-Watanabe M, TanabeM., “KEGG:integrating viruses and cellular organism, “. Nucleic Acids Res. 2021;49(D1):D545–D551. 10.1093/nar/gkaa970. 10.1093/nar/gkaa970 PMC7779016 33125081

[37] Jassal B, et al. The Reactome pathway knowledgebase. Nucleic Acids Res. 2020;48(D1):D498–D503. 10.1093/nar/gkz1031. 10.1093/nar/gkz1031 PMC7145712 31691815

[38] Ashburner M, et al., “Gene Ontology: tool for the unification ofbiology, “Nat Genet. 2000;25(1):25–9. 10.1038/75556. 10.1038/75556 PMC3037419 10802651

[39] Fisher RA., “On the interpretation of χ² from contingency tables, and the calculation of P, “JR Stat Soc. 1922;85(1):87–94. 10.2307/2340521.

[40] BenjaminiY Hochberg Y., “Controlling the false discovery rate: a practical and powerfulapproach to multiple testing, “J R Stat Soc Series B, 1995;57(1):289–300.

[41] Kaplan EL, Meier P., “Nonparametric estimation from in complete observations, “Journal of the American Statistical Association. 1958;53(282):457–81. 10.1080/01621459.1958.10501452.

[42] Cox DR. Regression models and life-tables. Journal of the Royal Statistical Society: Series B (Methodological). 1972;34(2):187–220.

[43] SondkaZ, Bamford S, Cole CG, Ward SA, Dunham I, Forbes SA., “TheCOSMIC Cancer Gene Census: describing genetic dysfunction across all humancancers,” Nature Reviews Cancer. 2018;18(11):696–705.10.1038/s41568-018-0060-1 10.1038/s41568-018-0060-1 PMC6450507 30293088

[44] Chakravarty D, Gao J, Phillips SM, Kundra R, Zhang H, Wang J, Rudolph JE, Yaeger R,Soumerai T, Nissan MH, Chang MT, Chandarlapaty S, Traina TA, Paik PK, Ho AL,Hyman DM, Baselga J, Taylor BS, Schultz N., “OncoKB: A Precision Oncology Knowledge Base,” JCO PrecisionOncology. 2017;1:PO.17.00011. 10.1200/PO.17.00011. 10.1200/PO.17.00011 PMC5586540 28890946

[45] Griffith M, Spies NC, Krysiak K, McMichael JF, Coffman AC, Danos AM, Ainscough BJ,Ramirez CA, Rieke DT, Kujan L, Barnell EK, Wagner AH, Skidmore ZL, Wollam AT,Liu CJ, Jones MR, Bilski RL, Lesurf R, Feng Y, Shah NM, Bonakdar M, Trani L,Matlock M, Ramu A, Campbell KM, Spies GC, Graubert AP, Gangavarapu K, EldredJM, Larson DE, Walker JR, Good BM, Wu C, Su AI, Dienstmann R, Margolin AA,Tamborero D, Lopez-Bigas N, Jacobsen A, McClellan M, Brown KM, Chmielecki J,Benson M, Chen K, Fehniger TA., “ CIViC is a community knowledgebase for expertcrowdsourcing the clinical interpretation of variants in cancer,” Nature Genetics, 49(2):170–174,2017. 10.1038/ng.3774. 10.1038/ng.3774 PMC5367263 28138153

[46] Liu Y, Sun J, Zhao M., “ ONGene: A literature-based database for human oncogenes, “ Journal of Genetics and Genomics. 2017;44(2):119–21. 10.1016/j.jgg.2016.12.004. 10.1016/j.jgg.2016.12.004 28162959

[47] Uhlén M, Zhang C, Lee S, Sjöstedt E, Fagerberg L, Bidkhori G, Benfeitas R, Arif M, Liu Z, Edfors F, Sanli K, von Feilitzen K, Oksvold P, Lundberg E, Hober S, Nilsson P, Mattsson J, Schwenk JM, Brunnström H, Glimelius B, Sjöblom T, Edqvist PH, Djureinovic D, Micke P, Lindskog C, Mardinoglu A, Pontén F. A., pathology atlas of the human cancer transcriptome, “Science. 2017;357(6352):eaan2507. 10.1126/science. 10.1126/science.aan2507 28818916

[48] Piñero J, Bravo À, Queralt-Rosinach N, Gutiérrez-Sacristán A, Deu-Pons J, Centeno E, García-García J, Sanz F, Furlong LI., “DisGeNET: a comprehensive platform integrating information on human disease-associated genes and variants,” Nucleic Acids Research. 2017.45(D1):D833–D839. 10.1093/nar/gkw943. 10.1093/nar/gkw943 PMC5210640 27924018

[49] Cancer Genome Atlas Research Network; Weinstein JN, Collisson EA, Mills GB, Shaw KR, Ozenberger BA, Ellrott K, Shmulevich I, Sander C, Stuart JM., “The Cancer Genome Atlas Pancancer analysis project, “Nat Genet. 2013:45(10):1113-20. 10.1038/ng.2764. PMID: 24071849; PMCID: PMC3919969. 10.1038/ng.2764 PMC3919969 24071849

[50] Pancancer analysis of whole genomes. Nature 578, 82–93, 2020. 10.1038/s41586-020-1969-6.

[51] Kirk S, Lee Y, Kumar P, Filippini J, Albertina B, Watson M, Rieger-Christ K, Lemmerman J. The Cancer Genome Atlas Lung Squamous Cell Carcinoma Collection (TCGA-LUSC) (Version 4) [Dataset], The Cancer Imaging Archive. 2018.

[52] Bradley J. Erickson and Felipe Kitamura,” Magician’s Corner: 9. Performance Metrics for Machine Learning Models,” Radiology: Artificial Intelligence 2021 3:3 10.1148/ryai.2021200126 PMC8204137 34136815

[53] Zhao, S., “Detection of LUAD-Associated Genes Using Wasserstein Distance in Multiomics Feature Selection,” Bioengineering, 12, 694, 2025. 10.3390/bioengineering12070694 10.3390/bioengineering12070694 PMC12292701 40722386

