# Supplementary material for: A novel statistical feature selection framework for biomarker discovery and cancer classification via multiomics integration
Source: BMC Med Res Methodol. 2025 Dec 17;26:11. doi: 10.1186/s12874-025-02713-z (PMC12822226; doi:10.1186/s12874-025-02713-z)
Supplement: Supplementary file 1 — Supplementary Material 1 [file 12874_2025_2713_MOESM1_ESM.pdf]

Supplementary Table S1: Classification Reports

Classification performance of the proposed late-fusion model (XGB + sDCFE + Deep Learning). Results are reported for the internal TCGA test set and the external PCAWG validation set. Metrics include precision, recall, F1-score, and support (number of samples).

TCGA Test (Pan-cancer)

Cancer Type	Precision	Recall	F1-score	Support
UCEC	1	0.94	0.97	35
THCA	1	1	1	100
STAD	0.988	1	0.99	83
PRAD	1	1	1	100
LUAD	0.990	1	0.995	103
LIHC	1	0.987	0.993	74
KIRP	0.982	0.966	0.97	58
COADREAD	1	1	1	76
LGG	0.990	1	0.995	103
BLCA	0.976	0.988	0.982	82
Accuracy	0.993	0.993	0.993	0.99
Macro avg	0.993	0.989	0.990	814
Weighted avg	0.993	0.993	0.993	814

PCAWG External Test (Pan-cancer)

Cancer Type	Precision	Recall	F1-score	Support
UCEC	0.96	0.90	0.93	51
THCA	1	0.94	0.97	48
STAD	0.88	0.77	0.82	30
PRAD	1	1	1	20
LUAD	1	0.8	0.89	90
LIHC	0.98	1	0.99	119

KIRP	0.99	0.98	0.99	185
COADREAD	0.73	0.98	0.84	55
LGG	1	1	1	48
BLCA	0.63	0.83	0.72	23
Accuracy	0.94	0.94	0.94	0.94
Macro avg	0.92	0.92	0.91	669
Weighted avg	0.95	0.94	0.94	669

Abbreviations: UCEC = Uterine Corpus Endometrial Carcinoma, THCA = Thyroid Carcinoma, STAD = Stomach Adenocarcinoma, PRAD = Prostate Adenocarcinoma, LUAD = Lung Adenocarcinoma, LIHC = Liver Hepatocellular Carcinoma, KIRP = Kidney Renal Papillary Cell Carcinoma, COADREAD = Colorectal Adenocarcinoma, LGG = Brain Lower Grade Glioma, BLCA = Bladder Urothelial Carcinoma.
