# Supplementary material for: A novel statistical feature selection framework for biomarker discovery and cancer classification via multiomics integration
Source: BMC Med Res Methodol. 2025 Dec 17;26:11. doi: 10.1186/s12874-025-02713-z (PMC12822226; doi:10.1186/s12874-025-02713-z)
Supplement: Supplementary file 2 — Supplementary Material 2 [file 12874_2025_2713_MOESM2_ESM.pdf]

Supplementary Table S2: Classification of Biomarker Novelty

The 82 intersected genes were evaluated across six curated resources (CIViC, COSMIC, OncoKB, ONGene, HPA, and DisGeNET). Based on their evidence profiles, genes were stratified into three categories: established biomarkers (n=12), emerging candidates (n=64), and novel genes (n=6**)**.

Gene Name	CIVIC	COSMIC	ONCOKB	ONGENE	HPA	DISGENT	Mean	Classification Category
**ACPP**					0	0	0	Novel candidate
**ACSM2A**					1	1	0.33	Emerging candidate
**ACSM2B**					1	1	0.33	Emerging candidate
**AFM**					1	1	0.33	Emerging candidate
**AHSG**					1	1	0.33	Emerging candidate
**ALB**			1		1	1	0.5	Established biomarker
**AMBP**					1	1	0.33	Emerging candidate
**APC2**					1	1	0.33	Emerging candidate
**APCS**					1	1	0.33	Emerging candidate
**APOC3**					1	1	0.33	Emerging candidate
**APOC4**					1	1	0.33	Emerging candidate
**ASGR2**					1	1	0.33	Emerging candidate
**ATP1B2**					1	1	0.33	Emerging candidate
**ATP2B2**					1	1	0.33	Emerging candidate
**BAIAP2L1**	1			1	1	1	0.66	Established biomarker
**C4BPA**					1	1	0.33	Emerging candidate
**C8G**					1	1	0.33	Emerging candidate
**CDHR5**					1	1	0.33	Emerging candidate
**CDX1**					1	1	0.33	Emerging candidate
**CFHR2**					1	1	0.33	Emerging candidate
**CREB3L3**					1	1	0.33	Emerging candidate
**CRP**					1	1	0.33	Emerging candidate
**F11**					1	1	0.33	Emerging candidate
**F11R**					1	1	0.33	Emerging candidate
**F9**					1	1	0.33	Emerging candidate
**FAM123A**					1	1	0.33	Emerging candidate
**FGFR4**	1	1		1	1	1	0.83	Established biomarker
**FGG**					1	1	0.33	Emerging candidate
**GFAP**					1	1	0.33	Emerging candidate
**GLYAT**					1	1	0.33	Emerging candidate
**GPA33**					1	1	0.33	Emerging candidate
**GPM6A**				1	1	1	0.5	Established biomarker
**GPRC5A**					1	1	0.33	Emerging candidate
**HAO1**					1	1	0.33	Emerging candidate
**HFE2**					0	0	0	Novel candidate
**HNF4A**					1	1	0.33	Emerging candidate
**ILDR1**					1	1	0.33	Emerging candidate
**ITGB6**					1	1	0.33	Emerging candidate
**ITIH1**					1	1	0.33	Emerging candidate
**ITIH3**					1	1	0.33	Emerging candidate
**KCNJ15**					1	1	0.33	Emerging candidate

**KCNJ16**					1	1	0.33	Emerging candidate
**KIF5C**					1	1	0.33	Emerging candidate
**KLK2**		1		1	1	1	0.66	Established biomarker
**KLK3**					1	1	0.33	Emerging candidate
**KLK4**					1	1	0.33	Emerging candidate
**KNG1**					1	1	0.33	Emerging candidate
**KRT18**	1				1	1	0.5	Established biomarker
**LMAN1L**					1	1	0.33	Emerging candidate
**LOC339674**					0	0	0	Novel candidate
**LRRTM2**					1	1	0.33	Emerging candidate
**MBL2**					1	1	0.33	Emerging candidate
**NAPSA**					1	1	0.33	Emerging candidate
**NAT8**					1	1	0.33	Emerging candidate
**NKX2-1**		1	1	1	1	1	0.83	Established biomarker
**NKX3-1**			1		1	1	0.5	Established biomarker
**OLIG2**	1		1		1	1	0.66	Established biomarker
**PAX8**	1	1	1	1	1	1	1	Established biomarker
**PEA15**					1	1	0.33	Emerging candidate
**PKP3**					1	1	0.33	Emerging candidate
**POU3F3**					1	1	0.33	Emerging candidate
**PTPRZ1**					1	1	0.33	Emerging candidate
**RAB25**					1	1	0.33	Emerging candidate
**RFX4**					1	1	0.33	Emerging candidate
**SCGB3A2**					1	1	0.33	Emerging candidate
**SERINC2**					0	0	0	Novel candidate
**SERPINA7**					1	1	0.33	Emerging candidate
**SFTA3**					0	0	0	Novel candidate
**SFTPA1**					1	1	0.33	Emerging candidate
**SFTPB**					1	1	0.33	Emerging candidate
**SHD**					1	1	0.33	Emerging candidate
**SLC13A1**					1	1	0.33	Emerging candidate
**SLC35E2**					1	1	0.33	Emerging candidate
**SLC44A4**					1	1	0.33	Emerging candidate
**SLC45A3**		1	1		1	1	0.66	Established biomarker
**SOX2OT**					0	0	0	Novel candidate
**TARP**					1	1	0.33	Emerging candidate
**TFR2**					1	1	0.33	Emerging candidate
**TG**					1	1	0.33	Emerging candidate
**TSHR**		1	1		1	1	0.66	Established biomarker
**UGT1A4**					1	1	0.33	Emerging candidate
**VTN**					1	1	0.33	Emerging candidate
