# Supplementary material for: A novel statistical feature selection framework for biomarker discovery and cancer classification via multiomics integration
Source: BMC Med Res Methodol. 2025 Dec 17;26:11. doi: 10.1186/s12874-025-02713-z (PMC12822226; doi:10.1186/s12874-025-02713-z)
Supplement: Supplementary file 3 — Supplementary Material 3 [file 12874_2025_2713_MOESM3_ESM.pdf]

## Supplementary Table S3: Survival Analysis of Novel Genes

Hazard ratios (HR) and p-values for six novel candidate genes across multiple cancer types. Significance levels are indicated as NS = not significant, borderline, sig = significant, strong sig, or very strong sig depending on p-value thresholds.

| Cancer | SOX2OT | ACPP | SFTA3 | SERINC2 | LOC339674 | HFE2 |
|---|---|---|---|---|---|---|
| **UCEC** | HR=1.27 p=0.096 (NS) | HR=0.98 p=0.897 (NS) | HR=1.28 p=0.114 (NS, low N) | HR=0.99 p=0.951 (NS) | HR=0.92 p=0.655 (NS) | HR=1.04 p=0.781 (NS, low N) |
| **THCA** | HR=1.51 p=0.085 (NS) | HR=0.88 p=0.622 (NS) | HR=0.63 p=0.046 (sig) | HR=0.64, p=0.051 (borderline) | HR=0.90, p=0.680 (NS) | HR=1.19, p=0.143 (NS, low N) |
| **STAD** | HR=1.04, p=0.643 (NS) | HR=1.03 p=0.737 (NS) | HR=1.03 p=0.635 (NS, low N) | HR=0.99, p=0.858 (NS) | HR=0.92, p=0.318 (NS) | HR=1.11, p=0.136 (NS, low N) |
| **PRAD** | HR=0.96, p=0.883 (NS) | HR=0.52p=0.021 (sig) | HR=1.06p=0.851 (NS, low N) | HR=0.66, p=0.113 (borderline) | HR=1.31, p=0.361 (NS) | HR=1.10, p=0.708 (NS) |
| **LUAD** | HR=0.88, p=0.087 (borderline) | HR=0.83, p=0.010 (sig) | HR=0.73, p=4.56e-07 (strong sig) | HR=1.09, p=0.303 (NS) | HR=1.08, p=0.304 (NS) | HR=0.92, p=0.220 (NS) |
| **LIHC** | HR=1.01, p=0.935 (NS) | HR=1.07, p=0.468 (NS) | HR=1.00, p=0.994 (NS, low N) | HR=1.13, p=0.197 (NS) | HR=1.27, p=0.003 (sig) | HR=0.81, p=0.006 (sig) |
| **KIRP** | HR=0.81, p=0.243 (NS) | HR=0.89, p=0.460 (NS) | HR=1.02, p=0.862 (NS, low N) | HR=0.76, p=0.020 (sig) | HR=1.51, p=0.001 (sig) | HR=1.30, p=0.050 (sig) |
| **COADREA** | HR=1.24, p=0.015 (sig) | HR=0.97, p=0.760 (NS) | HR=1.06, p=0.543 (NS, low N) | HR=0.90, p=0.300 (NS) | HR=1.41, p=0.00096 (sig) | HR=1.07, p=0.534 (NS, low N) |
| **LGG** | HR=0.98, p=0.847 (NS) | HR=1.26, p=0.002 (sig) | HR=0.91, p=0.384 (NS, low N) | HR=1.63, p=1.5e-07 (strong sig) | HR=0.45, p=6.8e-22 (very strong sig) | HR=0.71, p=5.6e-04 (sig) |
| **BLCA** | HR=1.15, p=0.054 (borderline) | HR=1.10, p=0.209 (NS) | HR=0.95, p=0.521 (NS, low N) | HR=0.83, p=0.008 (sig) | HR=1.00, p=0.959 (NS) | HR=0.86, p=0.061 (borderline, low N) |
